# The development of single-domain VHH nanobodies that target the *Candida albicans* cell surface

**DOI:** 10.1128/spectrum.04269-23

**Published:** 2024-10-07

**Authors:** Giuseppe Buda De Cesare, Frank M. Sauer, Anna Kolecka, Aimilia A. Stavrou, Theo C. Verrips, Teun Boekhout, Edward Dolk, Carol A. Munro

**Affiliations:** 1 Department of Microbiology and Molecular Genetics, The University of Texas Health Science Center at Houston, Houston, Texas, USA; 2 Institute of Medical Sciences, Foresterhill, United Kingdom; 3 QVQ, Utrecht, the Netherlands; 4 Previous Westerdijk Fungal Biodiversity Institute, Utrecht, the Netherlands; 5 GenDx, Utrecht, the Netherlands; 6 College of Sciences, King Saud University, Riyadh, Saudi Arabia; University of Michigan Michigan Medicine, Ann Arbor, Michigan, USA

**Keywords:** antibodies, *Candida albicans*, fungal cell wall

## Abstract

*Candida albicans* causes life-threatening invasive infections that are hard to diagnose and treat, with drug resistance leading to treatment failure. The goal of this study was to develop VHH (single variable domain on a heavy chain) nanobodies to detect drug-resistant infections. Llamas were immunized with a mixture of heat killed and fixed *C. albicans* cells of different morphologies. Llama lymphocyte RNA was used to generate phage display libraries that were tested for binding to *C. albicans* cells or cell wall fractions, and single antibody domains were isolated. The libraries were panned against echinocandin-resistant *C. albicans* isolates and counter-selected against echinocandin-susceptible isolates with the aim of isolating binding domains specific for antigens on drug-resistant cells. Thirty diverse VHH nanobodies were selected, and binding characteristics were assessed via dose-response ELISA. Binding was tested against a variety of *C. albicans* isolates and other *Candida* species, indicating that the VHHs were specific for *C. albicans*. The VHH nanobodies were sorted into four distinct groups based on their binding patterns. Two of the groups bound preferentially to the yeast cell poles and hyphae, respectively. Nanobody binding to *C. albicans* deletion mutants was tested by fluorescence microscopy and ELISA to identify the antigen targets. VHH19 nanobody, belonging to the largest group, recognized the Als4 adhesin. VHH14 antibody in the hyphae-specific group recognized Als3. None of the isolated VHH nanobodies was selective for drug-resistant clinical isolates. Our data indicate that this approach can generate valuable single-domain antibodies specific to *C. albicans* proteins.

**IMPORTANCE:**

The human fungal pathogen *Candida albicans* causes a range of diseases from superficial mucosal infections such as oral and vaginal thrush to life-threatening, systemic infections. Accurate and rapid diagnosis of these infections remains challenging, and currently, there are no rapid ways to diagnose drug-resistant infections without performing drug susceptibility testing from blood culture, which can take several days. In this proof-of-concept study, we have generated a diverse set of single domain VHH antibodies (nanobodies) from llamas that recognize and bind specifically to *C. albicans* cell surface. The nanobodies were classified into four groups based on their binding patterns, for example, cell poles or hyphae. Specific nanobodies were verified as recognizing the important adhesin Als4 or the hyphae associated invasin Als3, respectively. The data validate the approach that small VHH antibody domains hold future promise for diagnostic applications and as probes to study the fungal cell surface.

## INTRODUCTION

*Candida albicans* is a common constituent of the human mucosal mycobiota/microbiota that can colonize several niches in the body. *C. albicans* can cause infections that increase in severity according to the host’s immune state (1–3). The increasing resistance rates to current antifungals hampers the efficacy of therapy, complicating the treatment of candidiasis patients (4). From a diagnostics perspective, providing resolution at the species level (or even beyond) is important to guide therapy. Virulence and antifungal resistance vary between *Candida* species and even between strains of the same species (5, 6). Therefore, specific, accurate, and fast diagnosis of the causative agent of infections is crucial to rapidly start appropriate antifungal therapy. This is especially true in patients suffering from life-threatening candidiasis (7).

The discovery of heavy-chain-only antibodies in the serum of camelids was first reported by Hamers-Casterman et al. in 1993 (8). Single domain antibodies (VHHs) derived from this special class of antibodies paved the way for new possibilities in therapeutic and diagnostic applications. VHHs have unique features, such as a smaller size, that facilitate epitope specificity, tissue penetration, and shorter *in vivo* half-life (9). Moreover, high stability (10) and low immunogenicity (11, 12) are features suited for application in humans, such as therapeutics (13, 14) and cosmetics (15).

To date, the majority of monoclonal antibodies (mAbs), more recently isolated against *C. albicans,* were assessed as therapies, but not as diagnostics (16–19). In this work, the possibility of exploiting the VHH platform to generate diagnostic and research tools for *C. albicans* was explored. This study aimed to use phage display selection to isolate VHH domains that (1) bind to *C. albicans* cell surface and (2) detect surface epitopes specific to drug-resistant isolates. Knowing that *C. albicans* grows in a variety of morphologies dependent on environmental cues from host niches, we immunized llamas with *C. albicans* cells of different morphologies. Phage display technology and different bio-panning strategies were then used to select VHH domains. Specificity and affinity for echinocandin-resistant and sensitive *C. albicans* were assessed via dose-response ELISA. VHH binding to yeast cells and hyphae was also assessed. For target identification, deletion mutant screening was performed by fluorescence microscopy and ELISA, which enabled the identification of the targets for some of the antibodies.

## RESULTS

### Selection and validation of VHH binding to *C. albicans* whole cells

VHHs were identified using phage-display technology (20) and with the same strategy used for the anti-candidalysin VHH (21). Two *Llama glama* were independently immunized with a mixture of heat-killed and formaldehyde-inactivated *C. albicans* cells of different morphologies (yeast, germ tubes, and hyphae). The fungal cells were prepared by growing strain SC5314 in RPMI-1640 medium at 37°C with and without sub-inhibitory concentrations of caspofungin prior to fixing. After five booster injections with similar numbers of cells, blood samples were taken to verify the immune response to *C. albicans* by ELISA. Lymphocytes were subsequently extracted, total RNA was isolated and converted into cDNA, and the VHH genes were cloned into pMEK222 phagemid to obtain DNA libraries (Fig. S1). Two phage libraries, library 7 and library 11, were created, one from each llama.

Different bio-panning strategies were then performed to select VHH domains that bind to cell wall components specific to drug-resistant isolates. Two rounds of bio-panning were performed with phage display libraries 7 and 11, resulting in 16 output sub-libraries (Table 1). Each sub-library was the result of a different selection strategy using fixed cells or cell wall fractions from echinocandin-resistant isolates (Fig. S2). The first round of bio-panning was carried out by selecting the phage against fixed whole cells grown with or without caspofungin treatment. Cells were pooled from a mixture of three different echinocandin-resistant isolates (K063-3, B15_004476, and B12_007355_1) described in Buda De Cesare et al. (22). In the second round of bio-panning, counter selections were used with pooled cells from a mixture of echinocandin-susceptible isolates (SC5314, ATCC76615, and B17_008835) (Fig. S2). Pooled mixtures of cells from multiple isolates were used to avoid isolate-specific bias. Echinocandin-susceptible isolates were used in the counter selection with the aim to select clones with higher affinity for drug-resistant strains. For example, sub-library 1 was selected from a first round of bio-panning with fixed echinocandin-resistant cells and a second round of bio-panning with cell wall fractions from drug-resistant cells and a counter selection with fixed echinocandin-sensitive cells (Table 1). In general, caspofungin treatment of the fungal cells used for panning resulted in lower phage output (data not shown).

**TABLE 1:** Summary of the two rounds of biopanning and the counter selections used to generate 16 sub-libraries^*a*^

Selection (first round)	Selection second round (counterselection)	Phage output (first round)		Phage output (second round)		Sub-library #
		#7	#11	#7	#11	
R	CW^R^ (S)	10^6^	10^4^	10^2^	10^3^	1
R	CW^R^ + CAS (S)	10^6^	10^4^	10^3^	10^2^	2
R	R (S)	10^6^	10^4^	10^3^	10^5^	3
R	R + CAS (S)	10^6^	10^4^	10^2^	10^5^	4
R + CAS	CW^R^ (S)	10^5^	10^3^	10^3^	10^3^	5
R + CAS	CW^R^ + CAS (S)	10^5^	10^3^	10^3^	10^3^	6
R + CAS	R (S)	10^5^	10^3^	10^3^	10^5^	7
R + CAS	R + CAS (S)	10^5^	10^3^	10^4^	10^5^	8
R + CAS	CW^R^ (R)	10^5^	10^3^	10^4^	10^4^	9
R + CAS	CW^R^ + CAS (R)	10^5^	10^3^	10^4^	10^2^	10
R + CAS	R (R)	10^5^	10^3^	10^3^	10^5^	11
R + CAS	R + CAS (R)	10^5^	10^3^	10^4^	10^4^	12
R	CW^R^ + CAS	10^6^	10^4^	10^3^	10^3^	13
R	R + CAS	10^6^	10^4^	10^5^	10^5^	14
R + CAS	CW^R^ + CAS	10^5^	10^3^	10^3^	10^3^	15
R + CAS	R + CAS	10^5^	10^3^	10^4^	10^5^	16

^*a*^ In the first round of selection (column 1), biopanning was performed against fixed cells of pooled caspofungin-resistant isolates (R) with caspofungin treatment (+CAS) or untreated. In the second, biopanning (column 2) phage was selected based on binding to cell wall fractions (CWR) from echinocandin-resistant isolates that had been treated with caspofungin (+CAS) or untreated. Then, a counter selection was performed with echinocandin-susceptible (S) and -resistant (R) fixed cells, see letters in brackets in column 2. Phage output from the first round of biopanning was then used as input for the second round of selection. The phage titers from the outputs of each round of biopanning are presented for each of the original libraries, and the sub-library they generated is indicated in the last column.

**Fig 1:**
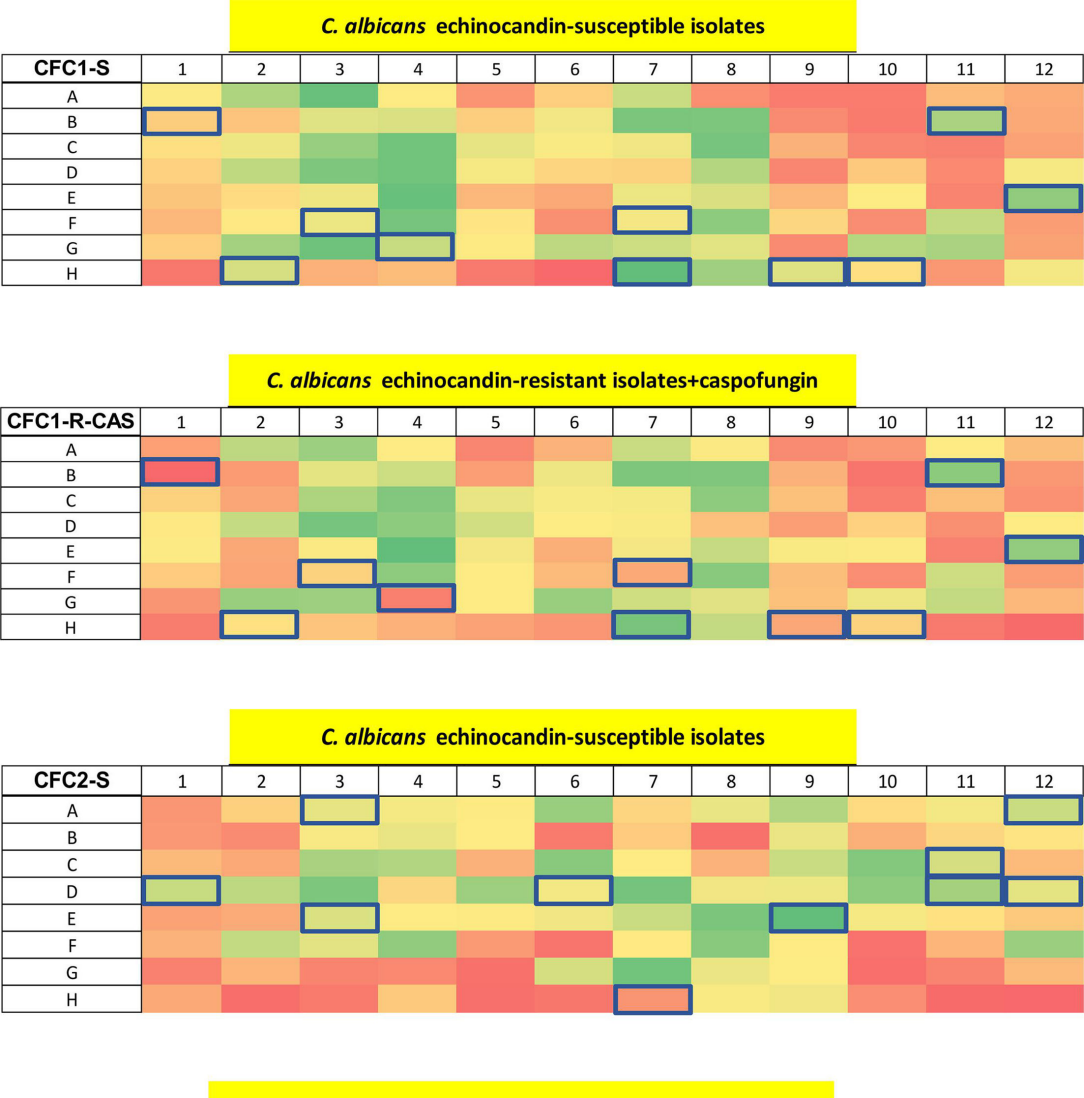
Whole cell ELISA of *C. albicans* isolates with periplasmic extracts containing the VHHs fused with the phage. Absorbance of ELISA performed with periplasmic extracts containing clones from libraries created after the second round of selection: clones from library 7 tested on echinocandin-susceptible (CFC1-S) and -resistant isolates treated with caspofungin (CFC1-R + CAS); clones from library 11 tested on echinocandin-susceptible (CFC2-S) and -resistant isolates treated with caspofungin (CFC2-R + CAS). The clones highlighted in blue were selected for further characterization based on differential binding between drug-resistant and drug-sensitive isolates with or without caspofungin treatment. The absorbance was calculated at 490 nm and subtracted from the background signal at 655 nm. The color code for the absorbance was set on the signal intensity of the negative controls (empty wells in H6 and H12; isotype control in well H5; and phage with empty pUR8100 plasmid in well H11). Zero percent indicates the signal intensity of the negative controls (red), and 100%, the highest signal in the plate (green). See Fig. S2 of supplementary materials for more information on the layout of the master plate.

Whole cell ELISAs were then carried out with the phage clones, using the same *C. albicans* isolates used for the second round of bio-panning selections, with or without caspofungin (Fig. 1; Fig. S2). Twenty phage clones were chosen for further characterization based on differential affinity toward the echinocandin-sensitive or -resistant isolates +/− caspofungin and on their predicted amino acid sequences (see below) (Table 2). In addition, 10 VHHs (CAW3-4 and CLY1 families) were included that had been selected following the same strategy described above except the second round of bio-panning was performed against whole cells of *C. albicans* SC5314. For this set, only the phages with the highest binding affinity were selected.

**TABLE 2:** VHH antibodies used in this work^*a*^

VHH #	VHH ID	MW (Da)	ε (L mol^−1^ cm^−1^)	Super-family
1	CFC2-E3	15,011	1.34	KGLEW
2	CFC2-D6	16,139	2.02	KEREG
3	CFC2-C11	15,877	2.40	KEREF
4	CFC2-D12	16,118	1.71	KEREG
5	CFC2-E9	16,099	1.96	KEREF
6	CFC2-A3	15,966	1.97	KEREF
7	CFC2-A12	16,084	2.05	KEREF
8	CFC1-B1	15,143	1.42	KQREL
9	CFC1-H2	15,212	1.42	KQREL
10	CFC1-E12	15,276	1.41	KQREL
11	CFC1-F7	15,011	1.80	KQREL
12	CFC1-H7	15,831	1.99	KEREF
13	CFC1-B11	14,652	1.47	KQREL
14	CAW3-A8	14,991	1.80	KQREL
15	CAW3-D5	15,274	1.77	KQREL
16	CAW4-D5	16,121	2.64	KEREF
17	CLY1-B2-A	16,100	1.96	KEREG
18	CLY1-B2-B	14,704	1.47	KQREL
19	CLY1-B2-C	16,154	2.04	KEREG
20	CLY1-C3-A	15,955	2.23	KEREG
21	CLY1-C3-B	15,979	2.22	KEREG
22	CLY1-H8	15,985	1.88	KEREF
23	CLY1-A11	14,718	1.46	KQREL
24	CFC1-F3	15,258	1.41	KQREL
25	CFC1-G4	15,195	1.42	KQREL
26	CFC1-H9	15,250	1.41	KQREL
27	CFC2-D1	15,218	1.42	KQREL
28	CFC1-H10	15,218	1.42	KQREL
29	CFC2-D11	14,718	1.46	KQREL
30	CFC2-H7	15,870	1.89	KEREF

^*a*^ CAW3 and CLY1 antibodies were previously isolated using *C. albicans* whole cells and cell wall extracts. The VHH IDs are based on the position of the clones on the plates shown in Fig. 1.

All 30 selected VHHs were sequenced to determine sequence diversity. The Kabat numbering scheme, which is used to identify the amino acid residues based upon CDRs and framework regions (FRs), was used to compare the sequences (Table 2) (23). Four different super-families were identified according to the FR2 motif: KQREL, KEREF, KEREG, and KGLEW. KQREL super-family was the largest one, with 50% (15 of 30) of the VHHs forming part of this group. Eight VHHs belong to KEREG family, and six VHHs were classified as KEREF family members. Only one VHH (#1) was part of the KGLEW super-family. The complementarity-determining regions, and in particular CDR3 of the isolated VHHs, had large sequence diversity.

After isolation by phage display and sequencing, the selected VHH sequences were cloned into pMEK222 vector (Fig. S1a), and the resulting constructs were expressed in *E. coli* TG1 cells, and VHHs were purified using immobilized metal affinity chromatography (IMAC) according to the protocol described in the methods section.

**Fig 2:**
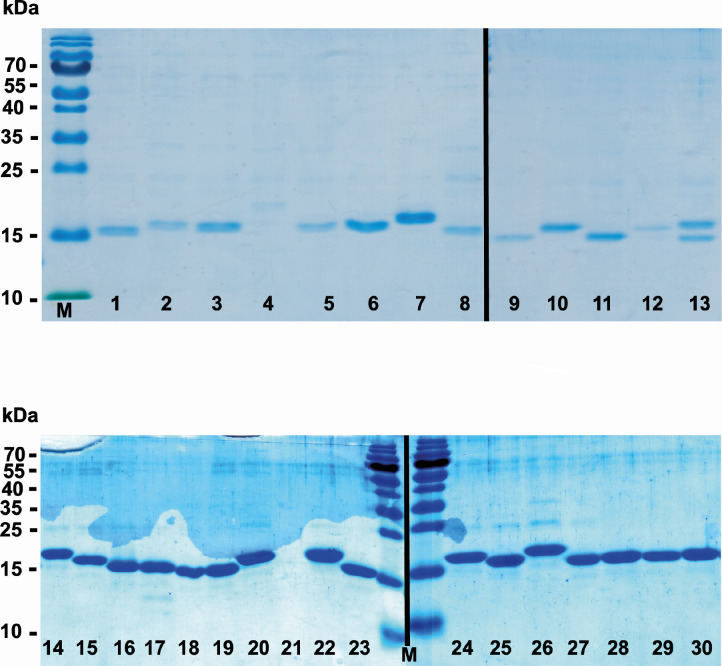
SDS-PAGE of VHH antibodies stained with Coomassie blue. The numbers in each lane correspond to the VHH number displayed in Table 2. M: PageRuler^TM^ prestained protein ladder.

To check the integrity of the extracted VHHs, SDS-PAGE analysis was performed (Fig. 2). As expected, the sizes of the selected VHHs were approximately 15 kDa. All the samples carried only negligible amounts of impurities after purification and dialysis, as shown by the absence or low concentrations of other protein bands. The Coomassie blue staining revealed slight differences in protein concentration for all the samples, except VHH4, 12, and 21, which were purified again (data not shown). VHH13 presented two bands of similar molecular weight (Fig. 2): the sample was then expressed and purified again, and the purity was checked by SDS-PAGE. The presence of only one clone was detected (data not shown).

### Dose-response ELISA to *C. albicans* whole cells

**Fig 3:**
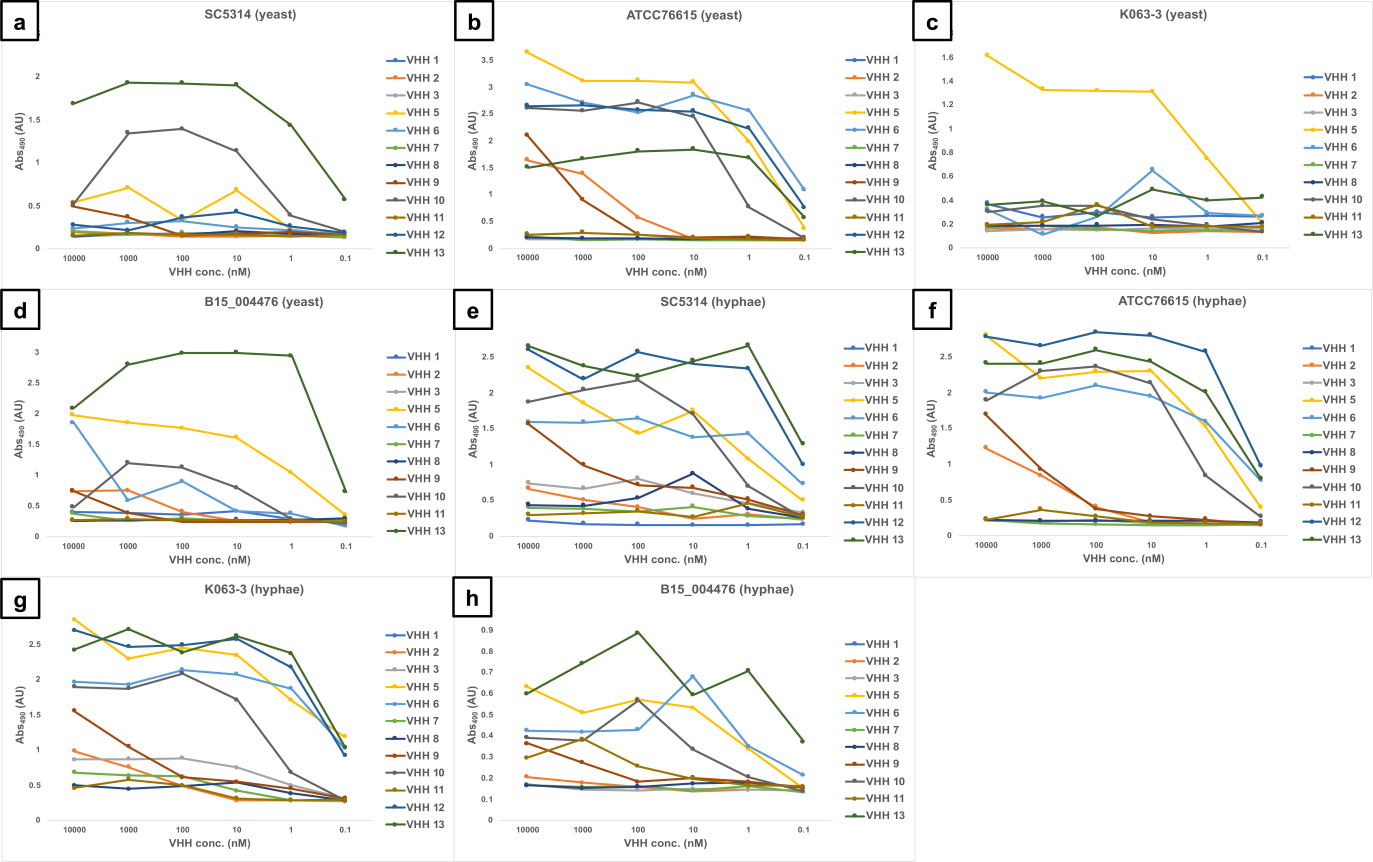
Dose-response ELISA of selected VHHs against echinocandin-resistant (K063-3 and B15_004476) and -susceptible (SC5314 and ATCC76615) isolates of *C. albicans*. Serial dilutions of the VHHs (from 10 µM to 0.1 nM) were tested against: (a) SC5314 yeast; (b) ATCC76615 yeast; (**C**) K063-3 yeast; (**d**) B15_004476 yeast; (e) SC5314 hyphae; (f) ATCC76615 hyphae; (g) K063-3 hyphae; and (h) B15_004476 hyphae.

VHH1-3, 5–13 were screened against *C. albicans* isolates in whole cell ELISA experiments using fixed yeast cells or hyphae (Fig. 3). VHH4 was omitted due to technical issues with its purification. The isolates used were the same as for the phage display bio-panning, with two caspofungin-susceptible (SC5314 and ATCC76615) and two -resistant (K063-3 and B15_004476) isolates. Binding was assessed by performing serial dilutions of the VHHs in a range between 10 µM and 0.1 nM. All the VHHs showed low nanomolar affinities, in the range of 10–0.1 nM, toward either yeast or hyphae. The binding signal from each antibody was different according to the strain and the morphology, potentially due to the genetic heterogeneity of the isolates that could influence epitope expression. For example, VHH1 showed high affinity for ATCC76615 yeast (Fig. 3b) and hyphae (Fig. 3f), SC5314 (Fig. 3e), and K063-3 hyphae (Fig. 3g). VHH1 did not give any significant signal with the other samples. Antibodies from the same super-family had different binding affinities. One example is given by #13 and #8 VHH nanobodies, which both belong to KQREL group. VHH13 had low nanomolar affinity (~0.1 nM) for all the samples assessed, whereas VHH8 had variable affinities dependent on the strain and cellular morphology. Another example was VHH5 and VHH6, part of KEREF family, which showed wide differences in affinity and binding properties according to the sample analyzed.

### ELISA on *C. albicans* cell wall mutants

**Fig 4:**
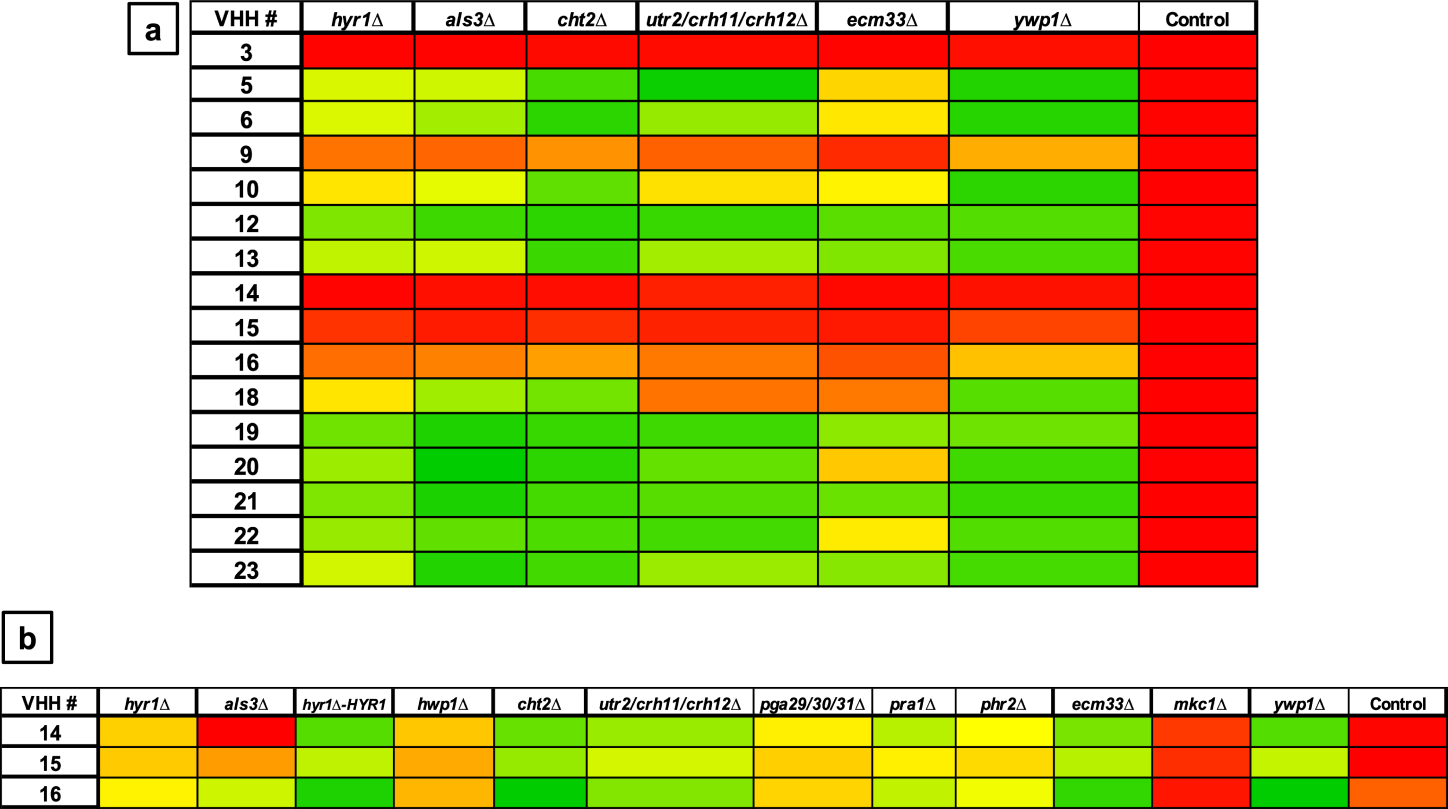
Whole-cell ELISAs with cell wall mutants and selected VHHs. The antibodies were tested against formaldehyde-inactivated cells, and the absorbance was measured at 490 nm (subtracting the background 655 nm) for yeast cells (a) and hyphae (b). Control is SC5314. The color code for the absorbance was set on the background signal given by the control (no cells). Zero percent indicates the signal intensity of the negative controls (red), and 100%, the highest signal in the plate (green). The measurements were the average of two technical replicates.

**Fig 5:**
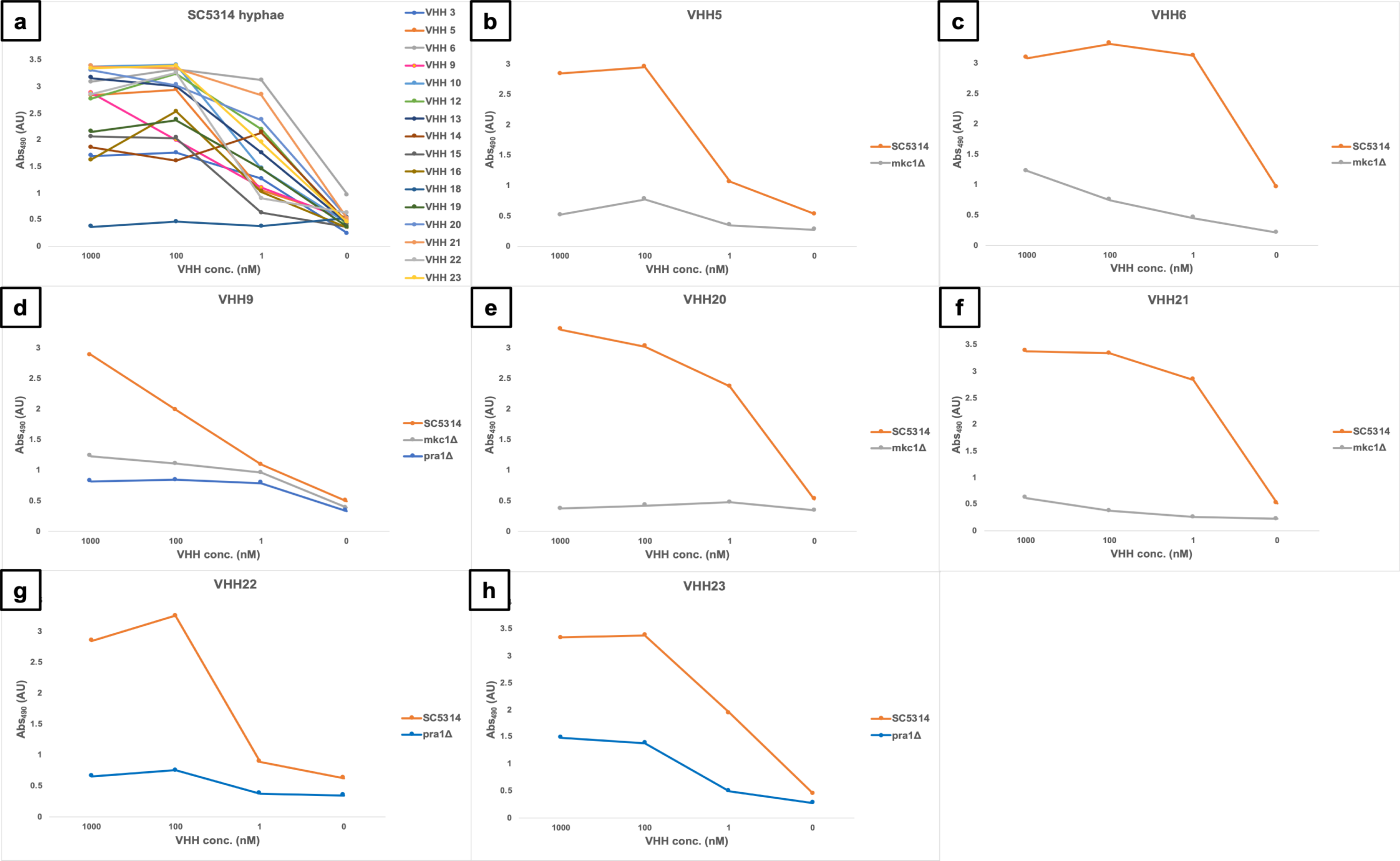
Dose-response ELISA of selected VHHs against filamentous forms of wild-type and cell wall mutants of *C. albicans*. Serial dilutions of the VHHs (1 µM, 100 µM, and 1 nM) were tested against (a) SC5314. Binding of the *mkc1*Δ mutant was tested with (b) VHH5, (c) VHH6, (d) VHH9, (e) VHH20, and (f) VHH21. Binding to the *pra1*Δ mutant was assessed for (d) VHH9, (g) VHH22, and (h) VHH23.

The next goal was to determine the specific cell surface epitope recognized by particular VHH nanobodies. Deletion mutants lacking specific cell wall proteins were screened in whole-cell ELISA experiments using fixed cells to identify differences in binding by selected VHHs (Fig. 4). The mutants were chosen based on the expression profiles of the proteins. They were either yeast or hyphae associated or known to be involved in caspofungin-induced cell wall remodeling (22). Mutants lacking specific cell wall proteins (Hyr1, Hwp1, Als3, Cht2, Utr2/Crh11/Crh12, Ecm33, Ywp1, Pra1, Phr2, and Pga29/30/31) or the cell integrity MAP kinase Mkc1 (known to strongly influence cell wall structure) were selected. Formaldehyde-fixed cells of the cell wall deletion mutants were used to screen VHHs for binding to yeast or filamentous morphologies. VHH14, 15, and 16 did not bind to yeast cells (Fig. 4a) but displayed binding to hyphae (Fig. 4b and 5a). VHH14,15,16 bound to hyphae of the reference strain SC5314 and most of the mutants, with some variation, but did not bind well to *mkc1Δ*, *hwp1*Δ, and *hyr1*Δ mutants (Fig. 4b and 5a). Binding of VHH14 to *als3*Δ was also strongly impaired (Fig. 4b).

To confirm the specific binding of the antibodies to hyphae, a dose-response ELISA was performed for SC5314 and the cell wall mutants (Fig. 5). All the VHHs displayed binding capacity for SC5314 hyphae, except for #18, which gave no signal (Fig. 5a). Five VHHs (#5, 6, 9, 20, and 21) had greatly reduced or absent binding to *mkc1*Δ mutant, which has several alterations in the cell wall and filamentation defects (24), compared with the wild-type isolate (Fig. 5b through f). VHH9, 22, and 23 had substantially lower affinities toward the hyphae of *pra1*Δ mutant, which has morphology defects (25), compared with SC5314 (Fig. 5d, g and h).

In general, some of the mutants, that is, *hyr1Δ*, *hwp1*Δ, *ecm33*Δ, and *och1*Δ (data not shown), gave low absorbance signals in ELISAs with most of the antibodies. This may be due to reduced coating capacity or antigen expression of the mutants. For this reason, no conclusions could be drawn from this part of the screen and the dose-response ELISAs with hyphae.

### Different immunolabeling patterns of the VHHs detected by fluorescence microscopy

To elucidate the immunolabeling patterns and identify the antigens recognized by the VHHs, fluorescence microscopy was performed on fixed yeast and hyphae, stained with calcofluor white. The immunolabeling controls are illustrated in Fig. S3 of Supplementary Material.

**Fig 6:**
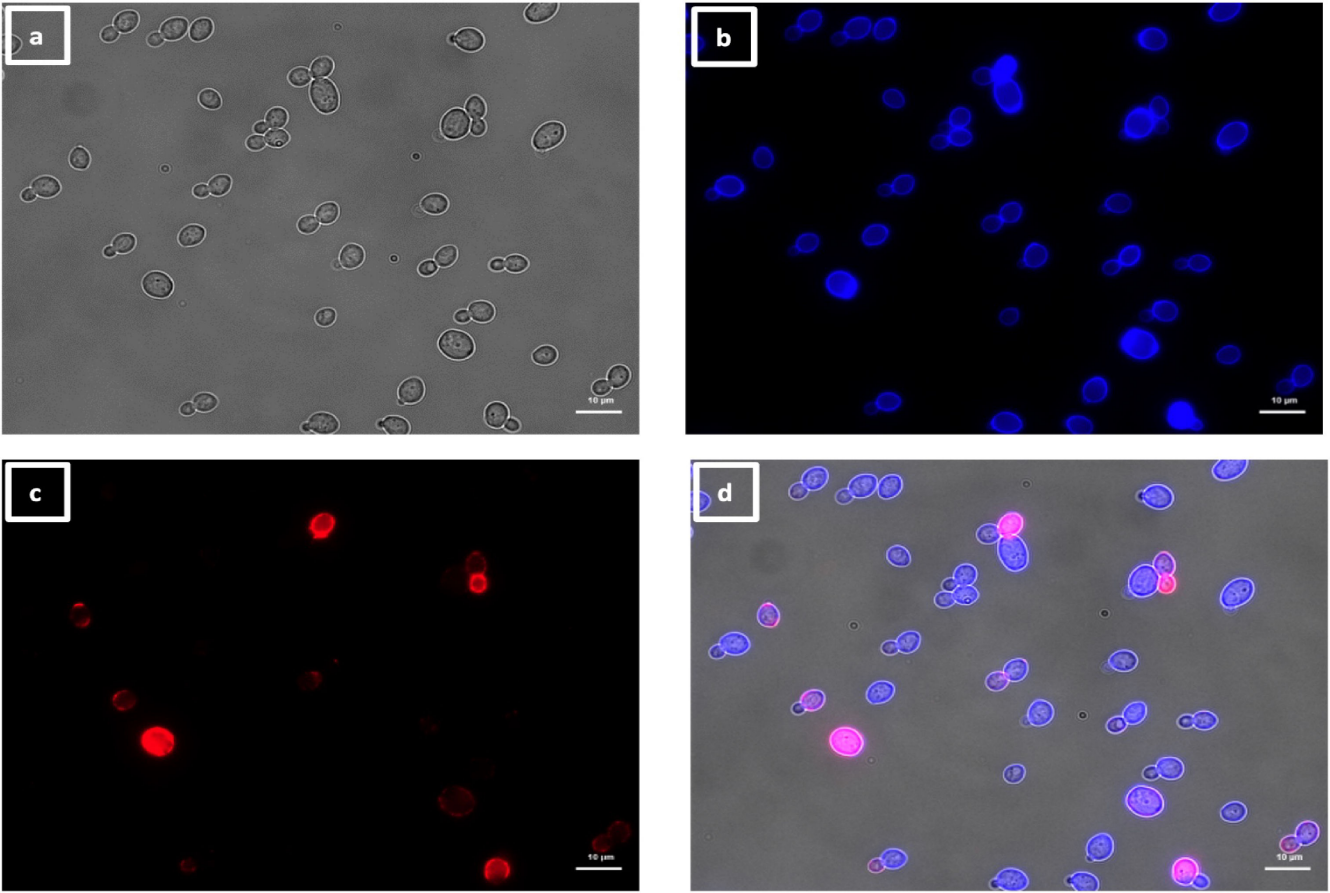
Immunolabeling of *C. albicans* yeast with VHH19. Yeast cells of *C. albicans* strain SC5314 were grown in YPD medium overnight at 30°C and fixed with formaldehyde. The samples were stained with calcofluor white and labeled with VHH19, and a rabbit anti-VHH polyclonal antibody and a goat anti-Rabbit IgG–Alexa Fluor 647–conjugated antibody for detection. Panels indicate: (a) DIC, (b) calcofluor white, (c) Alexa Fluor 647, and (d) merged view. Cells were imaged using a Zeiss Axio Imager M2 microscope.

**Fig 7:**
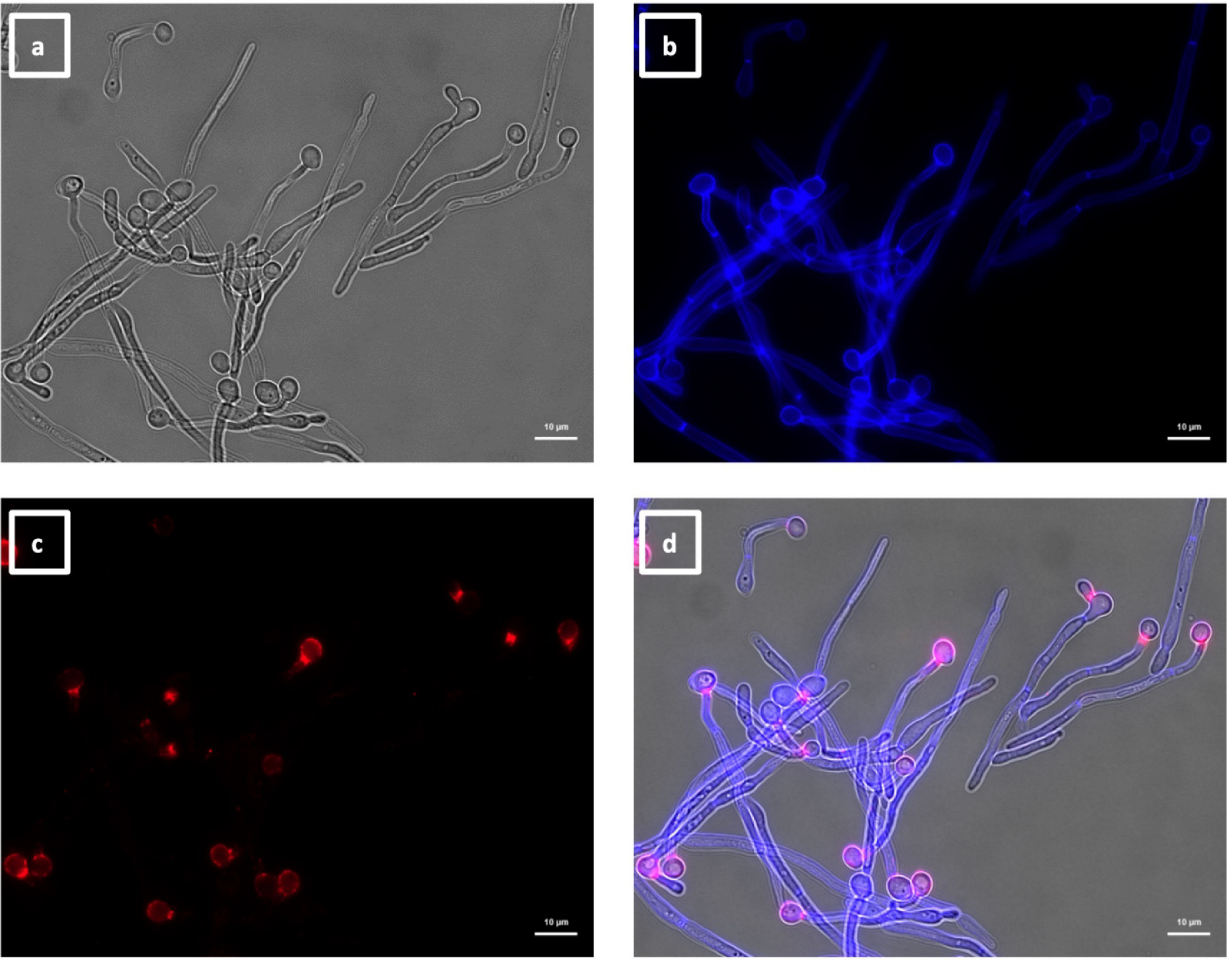
Immunolabeling of *C. albicans* hyphae with VHH19. Hyphae of *C. albicans* strain SC5314 were grown in RPMI-1640 medium at 37°C for 6 h and fixed with formaldehyde. The samples were stained with calcofluor white and labeled with VHH19, and a rabbit anti-VHH polyclonal antibody and a goat anti-Rabbit IgG–Alexa Fluor 647–conjugated antibody for detection. Panels indicate: (a) DIC, (b) calcofluor white, (c) Alexa Fluor 647, and (d) merged view. Cells were imaged using a Zeiss Axio Imager M2 microscope.

**Fig 8:**
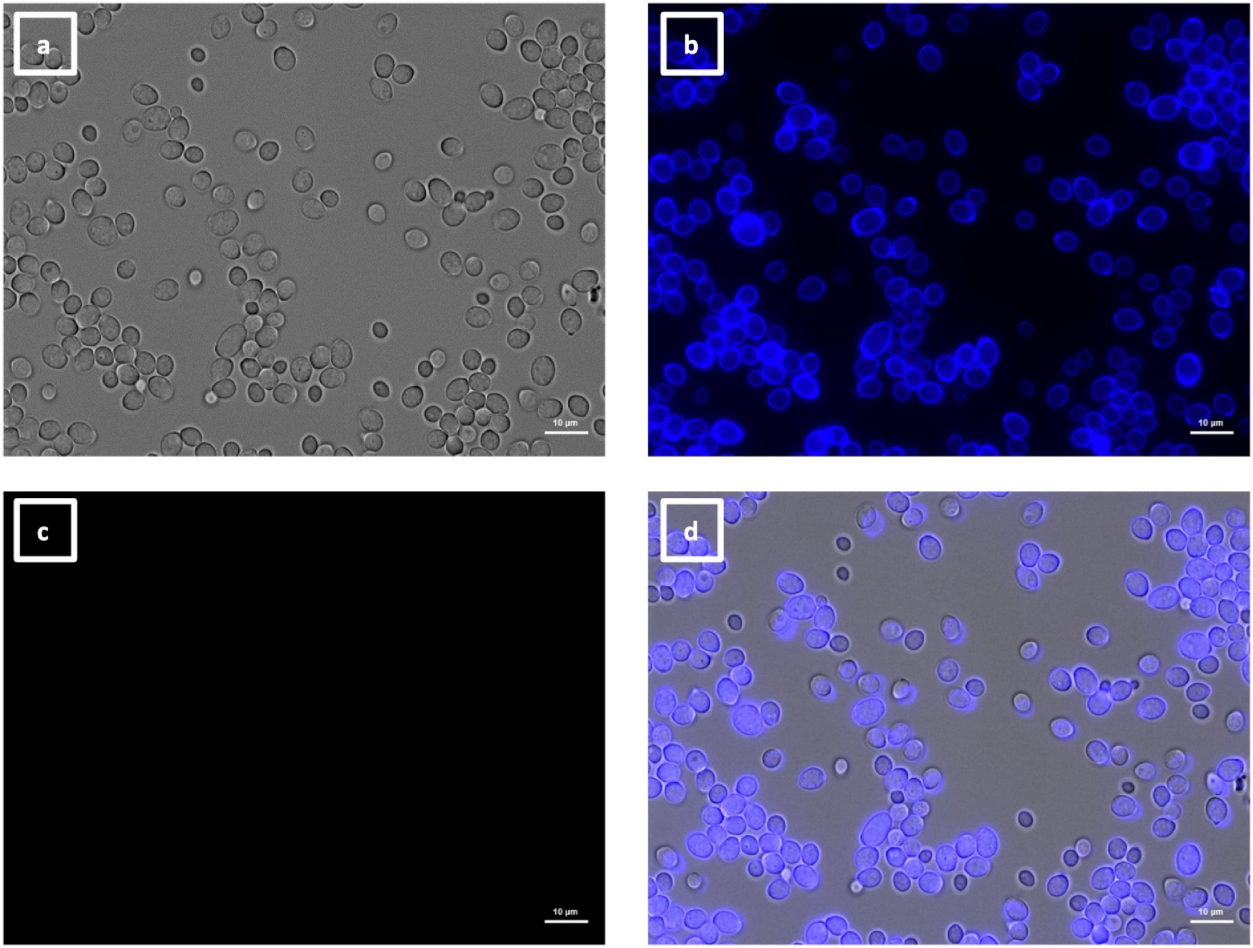
Immunolabeling of *C. albicans als4*Δ yeast with VHH19. Yeast cells of *C. albicans* strain *als4*Δ were grown in YPD medium overnight at 30°C and fixed with formaldehyde. The samples were labeled with calcofluor white and VHH19, and a rabbit anti-VHH polyclonal antibody and a goat anti-Rabbit IgG–Alexa Fluor 647–conjugated antibody for detection. Panels indicate: (a) DIC, (b) calcofluor white, (c) Alexa Fluor 647, and (d) merged view. Cells were imaged using a Zeiss Axio Imager M2 microscope.

**Fig 9:**
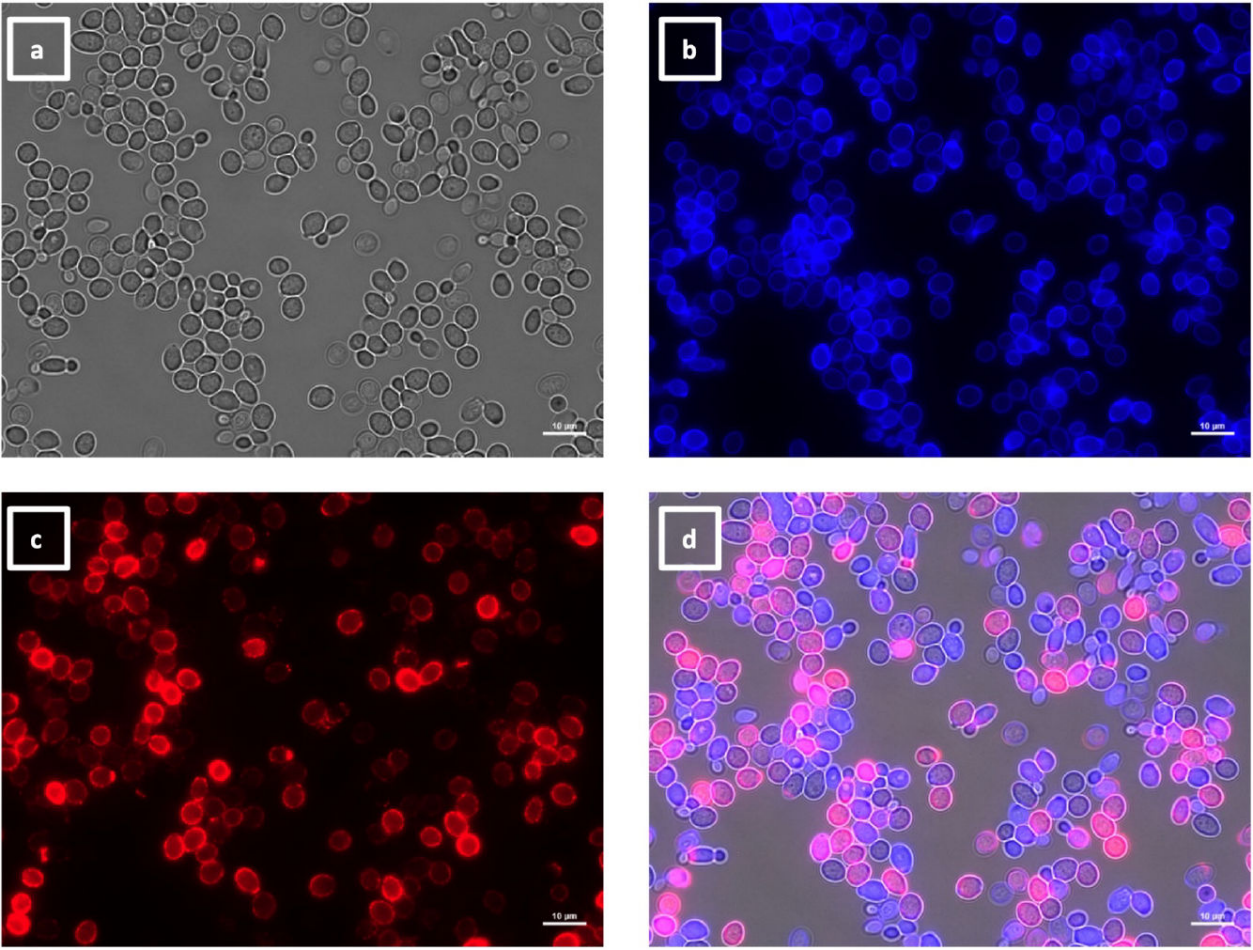
Immunolabeling of ketoconazole-treated *C. albicans* yeast with VHH19. Yeast cells of *C. albicans* strain SC5314 were grown in YPD medium with ketoconazole for 6 h at 30°C and fixed with formaldehyde. The samples were stained with calcofluor white and labeled with VHH19, and a rabbit anti-VHH polyclonal antibody and a goat anti-Rabbit IgG–Alexa Fluor 647–conjugated antibody for detection. Panels indicate: (a) DIC, (b) calcofluor white, (c) Alexa Fluor 647, and (d) merged view. Cells were imaged using a Zeiss Axio Imager M2 microscope.

The labeling patterns observed for the 30 VHHs were divided into four different groups. One group of 17 VHHs (i.e., 5, 6, 7, 8, 10, 11, 12, 13, 17, 18, 19, 20, 21, 22, 23, 29, and 30) immunolabeled a low number of SC5314 yeast cells within the same population exemplified by VHH19 binding (Fig. 6), with some yeast cells completely labeled over their entire surface, whereas others were only partially covered by the antibody. SC5314 hyphae were also immunolabeled but primarily around the interface between the mother yeast cells and the hyphal neck (Fig. 7), the binding pattern resembled the cellular localization pattern of Als4 previously shown by Hoyer and colleagues (26). Indeed, this group of VHHs, exemplified by VHH19, was not able to bind yeast or hyphae of an *als4*Δ mutant (Fig. 8). An increased number of immunolabeled cells could be observed upon ketoconazole treatment, which has been shown to increase Als4 expression (27) (Fig. 9).

**Fig 10:**
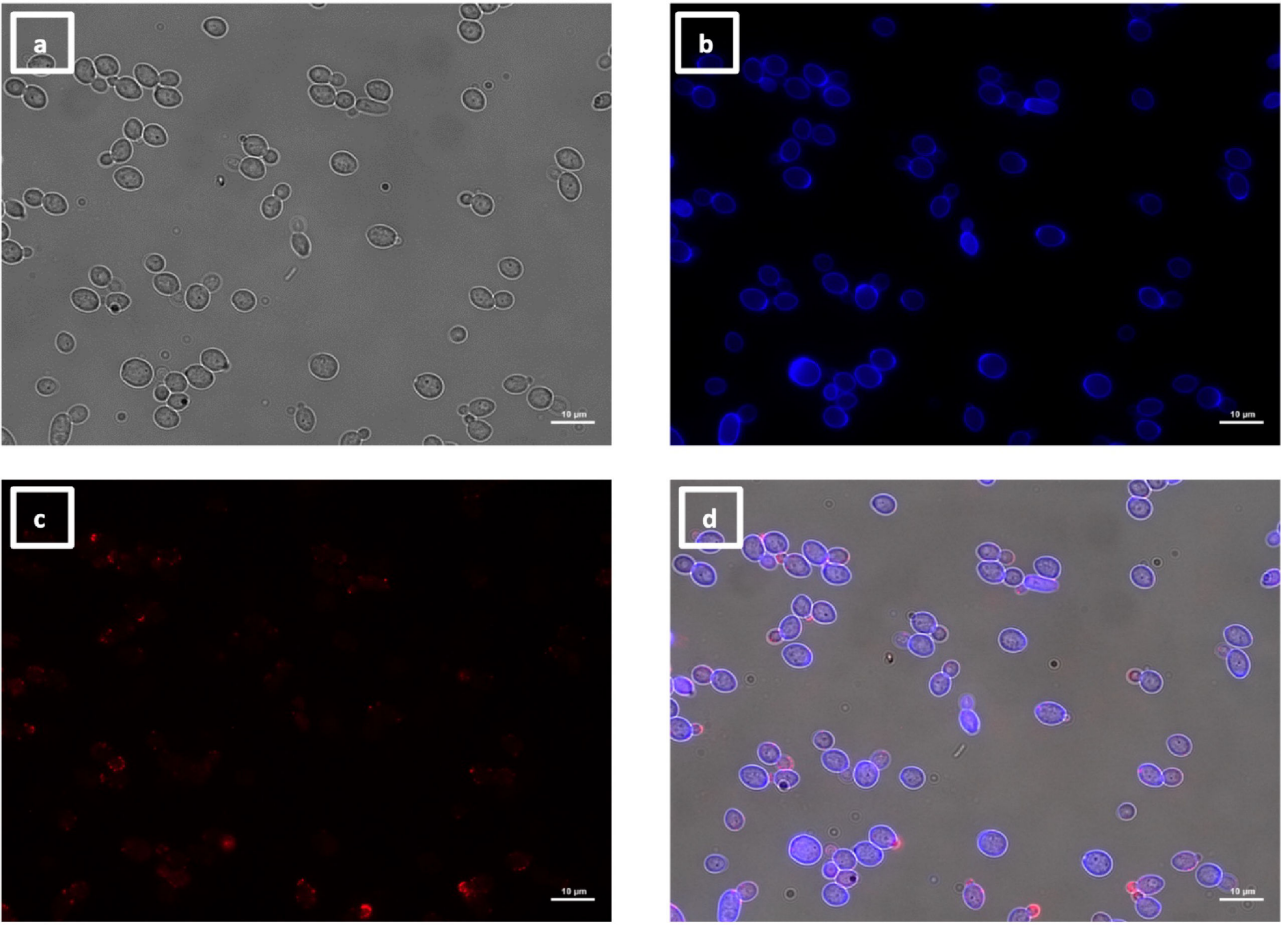
Immunolabeling of *C. albicans* yeast with VHH9. Yeast cells of *C. albicans* strain SC5314 were grown in YPD medium overnight at 30°C and fixed with formaldehyde. The samples were stained with calcofluor white and labeled with VHH9, and a rabbit anti-VHH polyclonal antibody and a goat anti-Rabbit IgG–Alexa Fluor 647–conjugated antibody for detection. Panels indicate: (a) DIC, (b) calcofluor white, (c) Alexa Fluor 647, and (d) merged view. Cells were imaged using a Zeiss Axio Imager M2 microscope.

**Fig 11:**
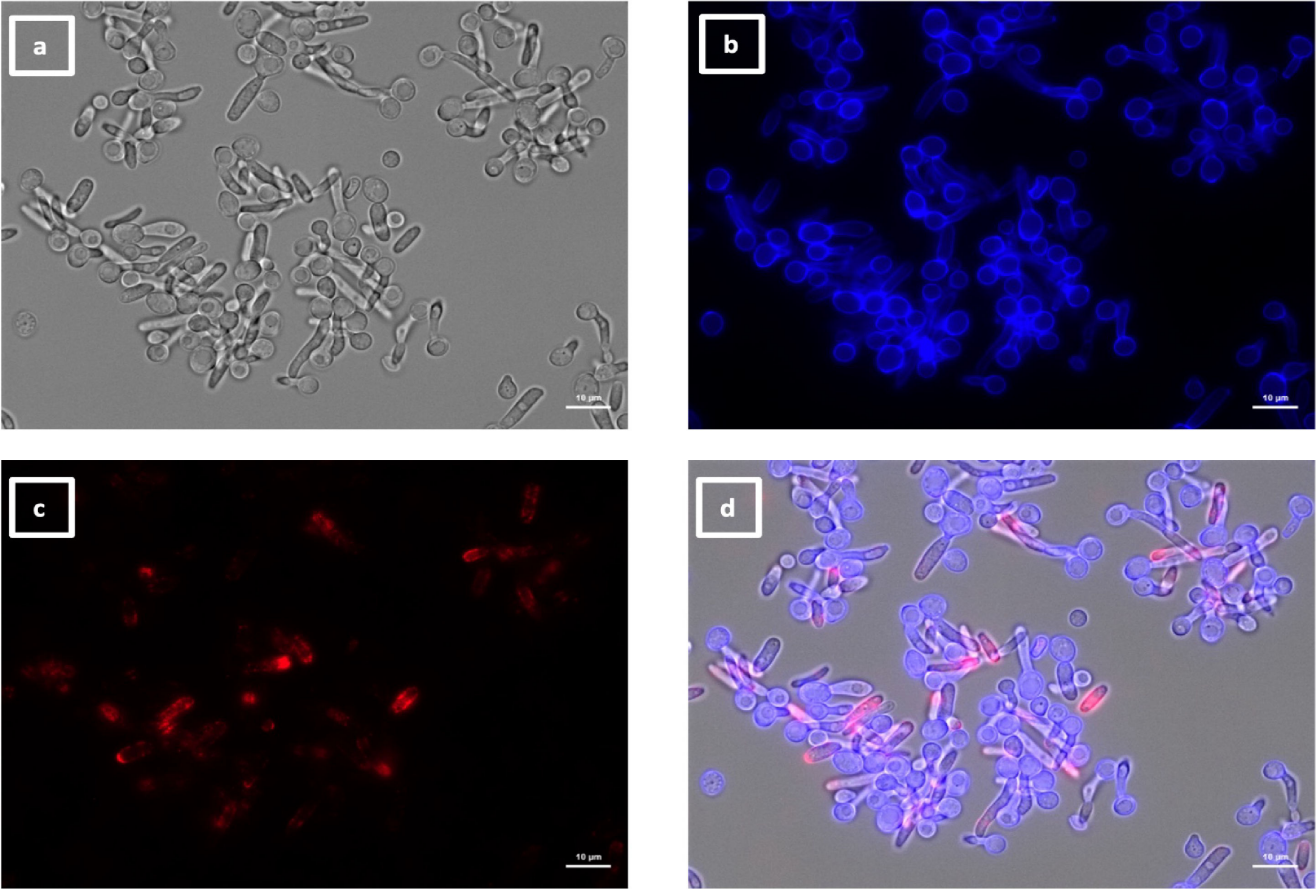
Immunolabeling of *C. albicans* pseudohyphae with VHH9. Pseudohyphae of *C. albicans* strain SC5314 were grown in YPD medium overnight at 30°C, starved for 24 h, transferred into RPMI-1640 medium at 30°C for 6 h, and then fixed with formaldehyde. The samples were stained with calcofluor white and labeled with VHH9, and a rabbit anti-VHH polyclonal antibody and a goat anti-Rabbit IgG–Alexa Fluor 647–conjugated antibody for detection. Panels indicate: (a) DIC, (b) calcofluor white, (c) Alexa Fluor 647, and (d) merged view. Cells were imaged using a Zeiss Axio Imager M2 microscope.

The second group of VHHs isolated was composed of six members (i.e., 9, 24, 25, 26, 27, and 28) binding to daughter cells budding from the mother cell, as shown in Fig. 10. The binding pattern varied slightly for different buds, probably due to spatial and temporal expressions of the antigen. This group of VHHs did not bind to hyphae (Fig. S4) but bound to pseudohyphae, specifically localized at the tip of the elongation (Fig. 11).

**Fig 12:**
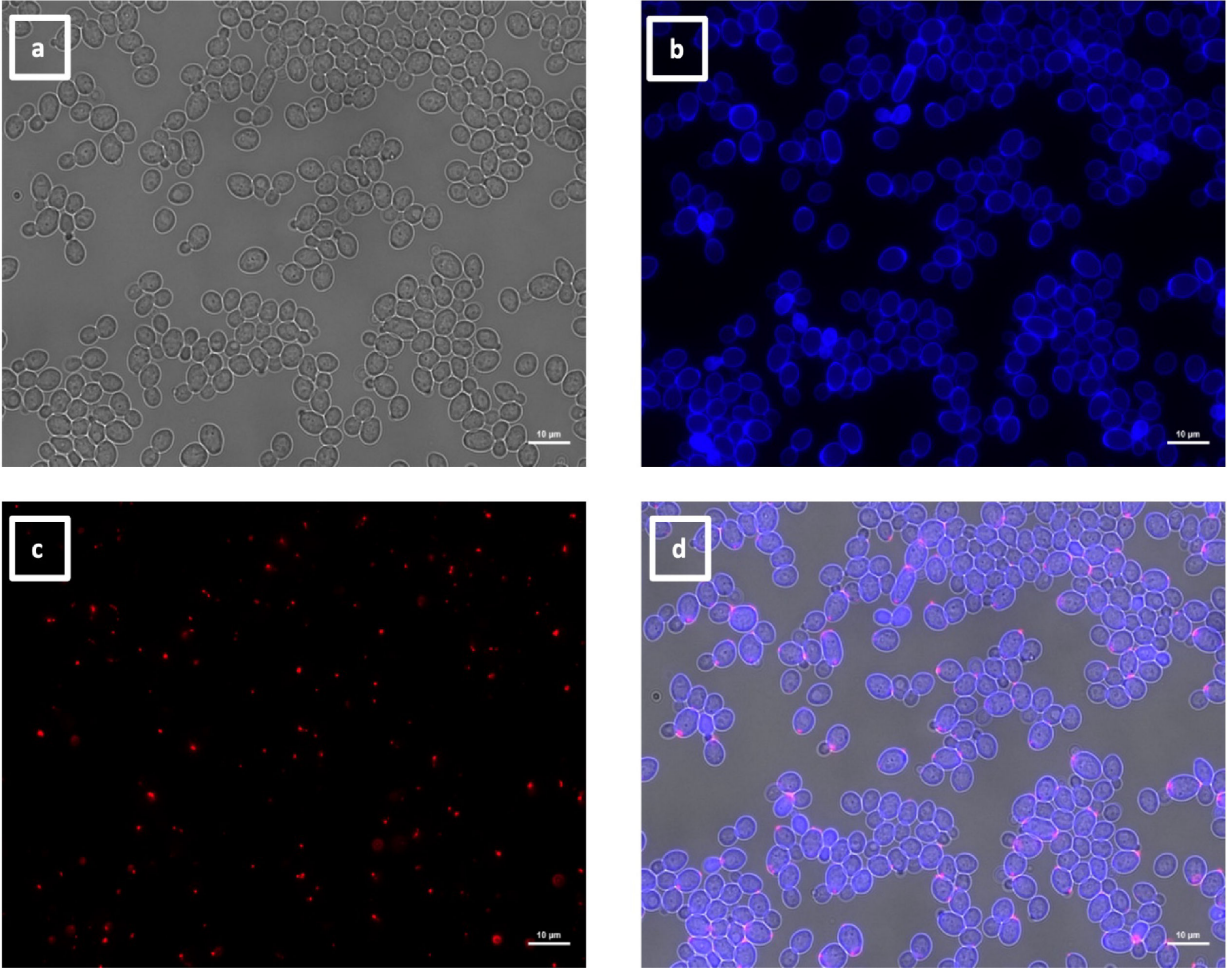
Immunolabeling of *C. albicans* yeast with VHH2. Yeast cells of *C. albicans* strain SC5314 were grown in YPD medium overnight at 30°C and fixed with formaldehyde. The samples were stained with calcofluor white and labeled with VHH2, and a rabbit anti-VHH polyclonal antibody and a goat anti-Rabbit IgG–Alexa Fluor 647–conjugated antibody for detection. Panels indicate: (a) DIC; (b) calcofluor white; (c) Alexa Fluor 647; and (d) merged view. Cells were imaged using a Zeiss Axio Imager M2 microscope.

A peculiar binding pattern was identified only for one antibody, VHH2, which bound to the poles of yeast cells. immunolabeling of SC5314 yeast cells showed defined spots on one or both extremities of the yeasts, but only about 40% of the cells were labeled, and the puncta were not always symmetrical (Fig. 12). VHH2 did not bind to SC5314 hyphae (Fig. S5).

**Fig 13:**
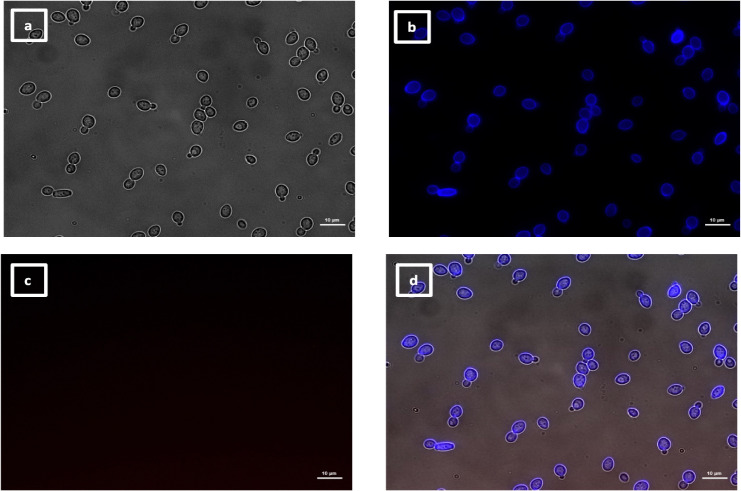
Immunolabeling of *C. albicans* yeast with VHH14. Yeast cells of *C. albicans* strain SC5314 were grown in YPD medium overnight at 30°C and fixed with formaldehyde. The samples were stained with calcofluor white and labeled with VHH14, and a rabbit anti-VHH polyclonal antibody and a goat anti-Rabbit IgG–Alexa Fluor 647–conjugated antibody for detection. Panels indicate: (a) DIC; (b) calcofluor white; (c) Alexa Fluor 647; and (d) merged view. Cells were imaged using a Zeiss Axio Imager M2 microscope.

**Fig 14:**
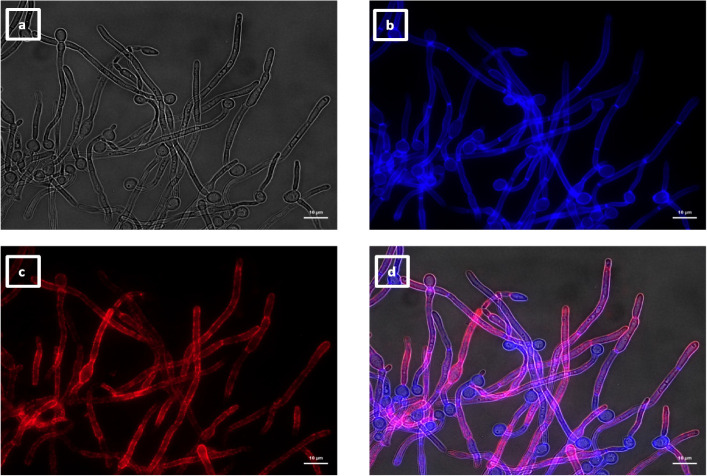
Immunolabeling of *C. albicans* hyphae with VHH14. Hyphae of *C. albicans* strain SC5314 were grown in RPMI-1640 medium at 37°C for 6 h and fixed with formaldehyde. The samples were stained with calcofluor white and and labeled with VHH14, and a rabbit anti-VHH polyclonal antibody and a goat anti-Rabbit IgG–Alexa Fluor 647–conjugated antibody for detection. Panels indicate: (a) DIC; (b) calcofluor white; (c) Alexa Fluor 647; and (d) merged view. Cells were imaged using a Zeiss Axio Imager M2 microscope.

**Fig 15:**
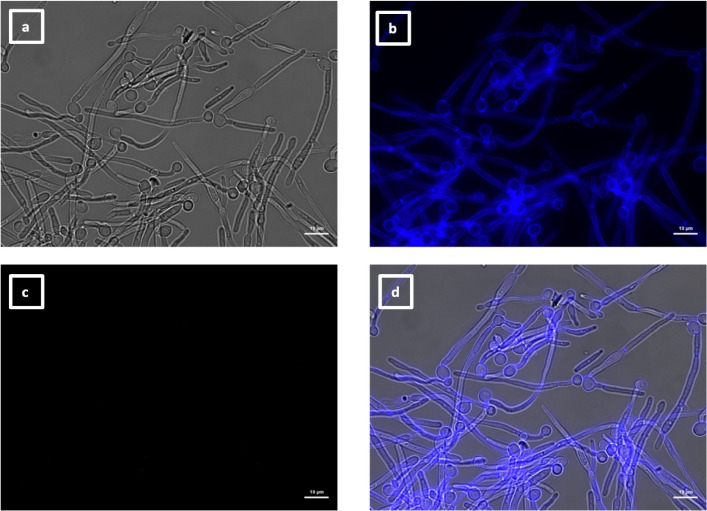
Immunolabeling of *C. albicans* als3Δ hyphae with VHH14. Hyphae of *C. albicans als3*Δ were grown in RPMI-1640 medium at 37°C for 6 h and fixed with formaldehyde. The samples were stained with calcofluor white and and labeled with VHH14, and a rabbit anti-VHH polyclonal antibody and a goat anti-Rabbit IgG–Alexa Fluor 647–conjugated antibody for detection. Panels indicate: (a) DIC; (b) calcofluor white; (c) Alexa Fluor 647; and (d) merged view. Cells were imaged using a Zeiss Axio Imager M2 microscope.

The last group of VHHs identified was specific for hyphae, as shown by immunolabeling of SC5314 yeast (Fig. 13) and hyphae (Fig. 14). In this group, there were two members binding an unknown antigen (#1, #4) and three members binding to Als3 protein (#14, #15, and #16). The specific recognition of the adhesin was shown by the reduction of binding to an *als3*Δ deletion mutant by ELISA (Fig. 4) and was particularly notable for VHH14 where there was a complete lack of binding to *als3*Δ observed by immunofluorescence microscopy (Fig. 15). VHH14 immunolabeling was rescued in the *als3*Δ + *ALS3* reintegrant strain, giving the same binding pattern as SC5314 (Fig. S6).

**Fig 16:**
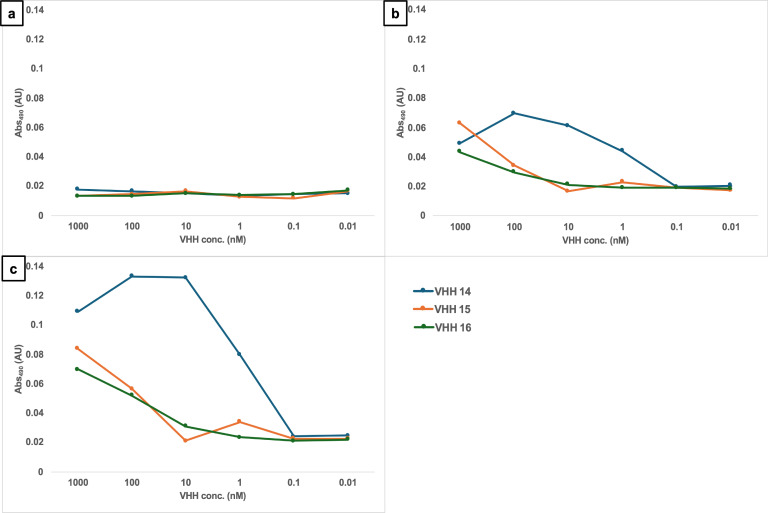
Dose-response ELISA of hyphae-specific VHHs against *C. albicans* hyphae. Serial dilutions of the VHH14 (blue), VHH15 (orange), and VHH16 (green) were tested against *C. albicans* SC5314 (a) yeast, (b) germ tubes, and (c) hyphae.

This evidence and the specificity for hyphae and not for yeast cells were also supported by dose-response ELISA experiments (Fig. 16). Whole cells of *C. albicans* yeast, germ tubes, and hyphae were incubated with antibodies belonging to this group, and only the germ tubes and hyphae gave a positive signal, albeit binding was weaker for VHH15 and VHH16.

### Specificity of VHH19 binding to Als4 measured by flow cytometry

**Fig 17:**
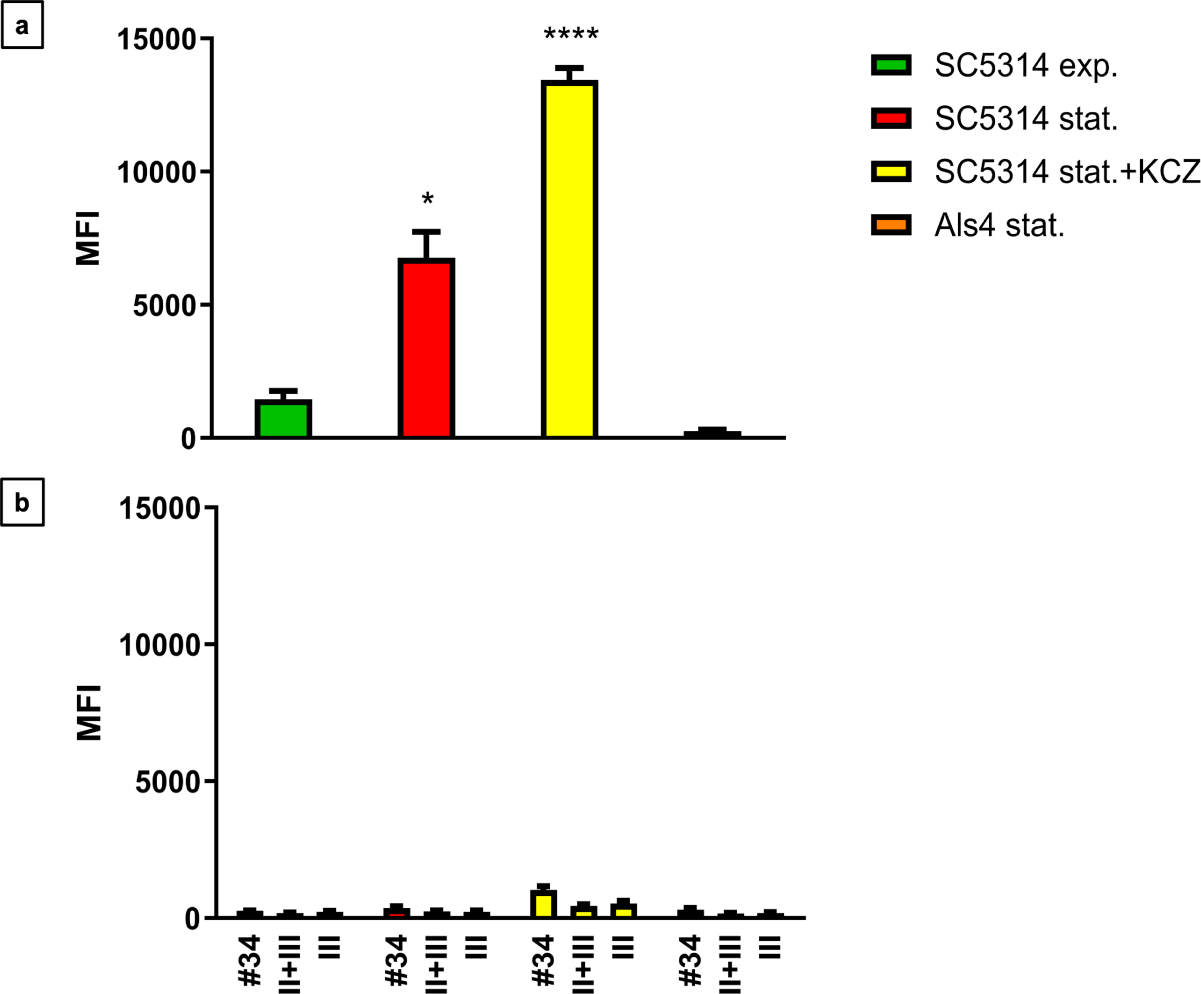
Flow cytometry of *C. albicans* yeast cells labeled with VHH19. The MFI was measured for *C. albicans als4*Δ mutant in stationery phase (Als4 stat.), SC5314 strain in exponential (SC5314 exp.), stationary phase (SC5314 stat.), or SC5314 treated with ketoconazole (SC5314 stat.+ KCZ) immunolabeled with (a) VHH19 and (b) isotype control (#34), no VHH control (II + III), and detection antibody-only control (III). The values represented three independent measurements, and the statistical significance was calculated with one-way ANOVA (*n* = 350,000 events analyzed, *P < 0.05, ****P < 0.00005).

Flow cytometry was performed to quantify VHH19 binding and confirm that VHH19 recognized the surface adhesin Als4. Cells of SC5314 were treated with ketoconazole, known to increase *ALS4* expression and compared with an *als4*Δ mutant. Stationary-phase SC5314 cells were treated with 19.13 µg/mL ketoconazole (KCZ) in YPD medium for 6 h at 30°C, fixed and labeled with VHH19. Comparisons were made to untreated SC5314 cells (exponential and stationary phase) and *als4*Δ mutant stationary phase cells. Binding of VHH was detected by flow cytometry. Little or no binding of VHH19 to stationary phase *als4*Δ mutant cells was detected with low median fluorescence intensity (MFI), confirming that VHH19 recognized the Als4 protein (Fig. 17a) comparable with the controls (Fig. 17b). As previously seen in the microscopy experiments, high numbers of immunolabeled cells were detected upon ketoconazole treatment, giving here the highest MFI. Wild-type cells in stationary phase showed a higher MFI than exponential phase cells, but not as much as the signal triggered by the drug treatment.

### Species specificity

**Fig 18:**
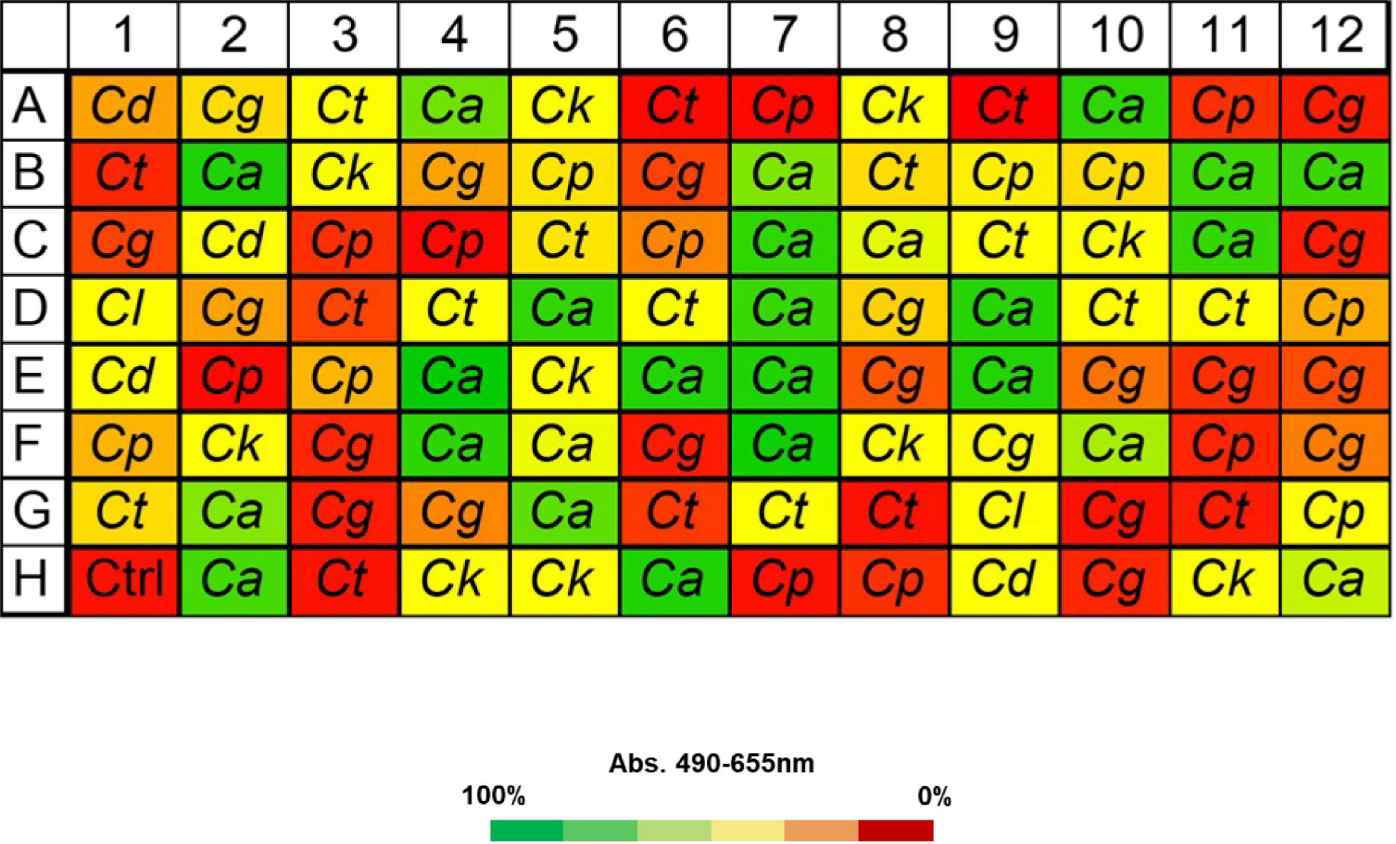
Whole-cell ELISA experiment to show species specificity of VHH19. VHH19 was tested for binding against *C. albicans (Ca*) and other *Candida* species: *C. dubliniensis (Cd), C. tropicalis (Ct), C. parapsilosis (Cp), C. glabrata (Cg), C. krusei (Ck),* and *C. lusitaniae (Cl*). Each well of the 96-well plates was coated with fixed fungal cells from each of the indicated species, and ELISA was performed with VHH19. According to absorbance values, VHH19 only recognized *C. albicans* isolates, indicated by the green squares. The absorbance was measured at 490 nm and subtracted from the background signal at 655 nm. The color code for the absorbance was set on the signal intensity of the empty well negative control (Ctrl): 0% indicates the signal intensity of the negative control (red) and 100%, the highest signal in the plate (green).

VHH19 from the KEREG superfamily was tested for specificity in recognizing *C. albicans* cells by ELISA. A blinded whole-cell ELISA experiment was performed on plates coated with different isolates of *Candida* species. Isolates of *C. albicans, Candida dubliniensis, Candida tropicalis, Candida parapsilosis, and Clavispora lusitaniae* (also known as *Candida lusitaniae*) were included. In addition, isolates of*, Nakaseomyces glabrata* and *Pichia kudriavzevii* (referred to as *Candida glabrata* and *Candida krusei* in this work, respectively) were also tested. The fungi were grown in YPD medium at 30°C overnight, formaldehyde-fixed, and tested for binding by VHH19. The results showed high specificity of VHH19 for *C. albicans* with strong binding to 22 of the 25 isolates tested and low-intermediate binding to the remaining three isolates. Low or no binding was detected to the other species tested (Fig. 18). In total, 25 *C*. *albicans* and 60 *Candida spp*. isolates were tested in duplicate, and no false positives or false negatives were detected.

## DISCUSSION

VHHs are single domain antibodies derived from heavy chain only, found in camelids, and their applications have a relatively recent history compared with conventional antibodies. Their particular features (e.g., stability at high temperatures, high solubility, and low toxicity) have been deployed in many fields such as diagnostics, imaging, and therapeutics (28). In this work, the use of various carefully selected immunogens and phage display selections was exploited with the aim to isolate VHH antibodies against drug-resistant isolates of *C. albicans,* which could potentially be used in the future to develop a rapid and effective diagnostic assay.

For isolation of the VHH, phage display technique was applied, using two libraries from llamas immunized with the *C. albicans* SC5314 strain in yeast, hyphae, and germ tube morphologies. Two rounds of selection were performed with whole cells and cell wall fractions from echinocandin-resistant and -susceptible isolates of *C. albicans*. A counter-selection was performed to increase the specificity of the isolated clones. The use of whole cells instead of single peptides for the selection eliminated concerns of misfolding of the proteins and enabled targeting of the extracellular portions of the proteins exposed on the cell surface. Moreover, the choice of the targets is unbiased, as the selection is left to the heterogeneity of the phage population. One of the limitations of this procedure is that the output is largely dependent on the isolates used for the bio-panning. Another disadvantage is that the antigen targets of the phage clones are unknown, and further analysis is required to identify and validate the specific targets. The phage sub-library outputs from the two libraries (summarized in Table 1) had different titers. Higher phage titer does not always correlate with higher heterogeneity in the phage population, as similar clones can be amplified multiple times. Moreover, whole cells bound more phage clones than the cell wall fractions, and this was expected as hyphae are very adhesive and more immunogenic than the isolated wall fractions, which may have epitopes that are not always in their native form.

To identify the VHHs that selectively bound to caspofungin-resistant isolates, a whole cell ELISA using the periplasmic extracts from the phage-infected cells was carried out (Fig. 1). Although no antibodies exclusively recognized drug-resistant isolates of *C. albicans*, 30 VHHs were isolated and sequenced (Table 2). The analysis of the amino acid sequences revealed four different super-families, based on Kabat numbering of the amino acid residues, and high variability in the CDRs, especially in CDR3.

The different VHHs were tested for binding to yeast and hyphae by whole cell ELISA, the majority could bind both, but some were found to recognize only hyphae (e.g., VHH1, 4, 14, 15, and 16). Binding to the reference strain SC5314 was compared with cell wall mutants to determine the targets of the VHHs. Some cell wall mutants such as *ecm33*Δ and *hyr1*Δ yeast cells, *hyr1*Δ, *hwp*1Δ, and *pga29/30/31*Δ filamentous forms had reduced capacity to coat the ELISA plates, probably due to adhesion defects. This resulted in false negative results.

Due to the variability of the ELISA results and the uncertainty given by the reduced coating capacity or the reduced expression of the antigens related to the mutations, the final evidence for antigen identification and antibody characterization was provided by fluorescence microscopy. Four different groups were identified based on the immunolabeling patterns.

The largest group immunolabeled yeast cells and the septum between the mother cell and a region of hyphal elongation proximal to the mother cell (Fig. 6 and 7). This group was exemplified by VHH19, and binding of VHH19 to Als4 protein was demonstrated by the absence of signal from *ALS4* deletion mutant yeast and hyphae (Fig. 8). These patterns were in line with the results observed in the work from Coleman et al. (29), where an anti-Als4 mAb was able to bind to *C. albicans* as well as to *C. tropicalis*; this group of VHHs did not, however, recognize *C. tropicalis* (Fig. 18). Moreover, VHH19 was also able to detect changes in Als4 expression on the cell wall, as displayed by the increased immunolabeling of ketoconazole-treated cells (Fig. 9), in agreement with the observation by Liu et al. (27). A quantitative validation of the results from Als4 binder VHH19 was also provided by flow cytometry analysis, which confirmed the differences observed for the drug treatment and Als4 expression during the growth phase (Fig. 17).

The second class of antibodies with an identified antigen comprised of hyphae-specific binders. One of these, VHH14, was demonstrated to bind to Als3, an adhesin associated exclusively with the hyphal cell surface (30). The antigen specificity of VHH14 was confirmed by fluorescence microscopy (Fig. 15) and ELISA (Fig. 16) using an *ALS3* deletion mutant. VHH1 and 4 belong to the same class of hyphae-specific antibodies but did not have any specificity for the Als3 protein. Mutants for other hyphae associated cell wall proteins were screened, without any success in the identification of the target antigen (data not shown).

The last two groups of VHHs comprised VHHs recognizing cell buds and cell poles (Fig. 10 and 12). The former class showed specificity not only for budding cells but also for pseudohyphae (Fig. 11). The latter class of antibodies bound in concentrated spots located on the poles of the cells (Fig. 12). The target antigens for both classes of antibodies are unknown. For the VHHs targeting the cell buds, a similar binding pattern was observed for the anti-Csa1 mAb in *S. cerevisiae* (31). Other similar patterns were observed for Rga2 and Cdc42 fluorescently tagged cells (32, 33). GFP-tagged Pea2 cells of *S. cerevisiae* presented comparable patterns, found in the database of fluorescent proteins CYCLoPs (https://thecellvision.org/cyclops/) created by Chong and colleagues (34). Those proteins are required for the establishment of polarity as well as pseudohyphal growth (35); hence, the antigen of this group may have a possible role in polarity and morphogenesis. For the other group of VHHs, recognizing the cell poles, antigens with a similar role could be hypothesized, as Nbp1 and Nud1 as GFP-tagged proteins presented similar patterns in *S. cerevisiae*. In fact, both are intracellular proteins involved in spindle pole organization and microtubular scaffold (36, 37).

Considering all these data, including the nanomolar affinity and the specificity for *C. albicans*, VHHs are a suitable research tool for *C. albicans* and can be developed in the future as diagnostics to detect candidiasis. Future work could include the development of bispecific VHHs to improve specificity and sensitivity. This approach did not lead to the identification of a nanobody binding selectively to drug-resistant clinical isolates, suggesting that there are no cell wall-localized antigens that are unique to the drug-resistant isolates used in this study, confirming the findings of Buda De Cesare et al. (22). Future diagnostics based on detection of cell surface proteins would require extensive pre-clinical and clinical testing to ensure the target of the diagnostic was conserved and the nanobody capable of detecting clinical isolates with different genetic backgrounds.

## MATERIAL AND METHODS

### Strains and growth conditions

The fungal and bacterial strains used in this study are listed in Table 3. Fungal cells were stored in 25% glycerol at −80°C and re-cultured in YPD agar (1% [wt/vol] yeast extract, 2% [wt/vol] mycological peptone, 2% [wt/vol] glucose, 2% [wt/vol] agar). For overnight cultures, unless otherwise indicated, a single colony of each strain was inoculated into YPD broth (1% [wt/vol] yeast extract, 2% [wt/vol] mycological peptone, 2% [wt/vol] glucose) and incubated overnight at 30°C with shaking at 200 rpm. For induction of hyphae, cells were grown in RPMI-1640 modified medium (50% [wt/vol] RPMI-1640, pH 0.8–1.5; 1.65M MOPS buffer, pH 7.2; 3.6% [wt/vol] glucose; 4.2 mM L-glutamine) with 20% Fetal Calf Serum (FCS) added, at 37°C for 6 h with 100 rpm shaking. Bacterial cells were stored in 50% glycerol at −80°C. *Escherichia coli* was usually grown at 37°C in LB medium (Luria Bertani; 1% [wt/vol] bacto-tryptone, 0.5% [wt/vol] bacto-yeast extract, 1% [wt/vol] NaCl, 1.8% [wt/vol] agar). To produce VHHs in *E. coli*, cells were cultured in 2xYT medium (1% [wt/vol] bacto-tryptone, 0.5% [wt/vol] bacto-yeast extract, 1% [wt/vol] NaCl, 2% [wt/vol] glucose supplemented with 100 µg/mL ampicillin or 25 µg/mL kanamycin) at 37°C. Unless otherwise stated, all chemicals were supplied by Sigma-Aldrich Co.

**TABLE 3:** Strains used in this study

Species	Strain ID	Description	Genotype	Reference
*C. albicans*	SC5314	Clinical isolate	*wild type*	(38)
*C. albicans*	CBS8758	Clinical isolate	*wild type*	(39)
*C. albicans*	ATCC2091	Serotype A	*unknown*	(40)
*C. albicans*	ATCC76615	Clinical isolate	*unknown*	(41)
*C. albicans*	B17_009053	Clinical isolate	*unknown*	(22)
*C. albicans*	B17_008835	Clinical isolate	*unknown*	(22)
*C. albicans*	K063-3	Derived from SC5314	*GSC1 (S645Y)/ GSC1 (S645Y)*	(42)
*C. albicans*	B15_004476	Clinical isolate	*unknown*	(22)
*C. albicans*	B12_007355_1	Clinical isolate	*GSC1 (R1361G)/ GSC1 (R1361G)*	(22)
*C. albicans*	PEG10_91	Clinical isolate	*unknown*	(22)
*C. albicans*	B15_012740	Clinical isolate	*unknown*	(22)
*C. albicans*	NGY205	*ch1∆*	*ch1*Δ*::hisG/och1*Δ*::hisG*	(43)
*C. albicans*	NGY358	*ch1∆-OCH1*	*ch1*Δ*::hisG/och1*Δ*::hisGRPS1/rps1Δ::CIp10-OCH1*	(43)
*C. albicans*	1843	*als3∆*	*iro1-ura3*Δ::*λimm434/iro1-ura3*Δ::*λimm434; als3la*Δ*/als3saΔ-URA3*	(44)
*C. albicans*	CAQTP178U	*als3Δ-ALS3*	*ura3*Δ*::λimm434::URA3-IRO1 als3::ARG4::ALS3 arg4::hisG his1::higG*	(45)
*C. albicans*	*hyr1Δ*	*hyr1Δ*	*Δura3::λimm434/Δura3::λimm434 hyr1::hisG::URA3::hisG/ hyr1::hisG::URA3::hisG*	(46)
*C. albicans*	C1637	*hyr1Δ-HYR1*	*ura3Δ::imm434/ura3Δ::imm434hyr1::hisG/ hyr1:: hisGRPS1/rps1Δ::CIp10-HYR1*	(16)
*C. albicans*	CAH7-1A1E2	*hwp1Δ*	*hwp1::hisG/hwp1::hisG eno1::URA3*	(47)
*C. albicans*	DSY1768	*cht2∆*	*cht2::hisG-URA3-hisG/cht2::hisG*	(48)
*C. albicans*	GPY102	*utr2/crh11/crh12 triple mutant*	*utr2::hisG/utr2::hisG; crh12::hisG/crh12::hisG; crh11::hisG/crh11::hisG-URA3-hisG*	(49)
*C. albicans*	pga29,30,31∆	*pga29,30,31∆ triple mutant*	*pga29/pga30/pga31*Δ*::ARG4/pga29/pga30/pga31*Δ*:: HISrps1*Δ*/RPS1::CIp10*	Ibe & Munro, unpublished (C. Ibe Phd Thesis, University of Aberdeen)
*C. albicans*	*pra1∆*	*pra1∆*	*pra1::HIS/pra1::ARG +CIp10*	(50)
*C. albicans*	584	*ywp1∆*	*ywp1*Δ* : : ARG4/ywp1*Δ : : *URA3-dpl200 his1 : : hisG/his1 : : hisG*	(51)
*C. albicans*	CFM-4	*phr2∆*	*phr2*Δ*::hisG/phr2*Δ*::hisG ura3* Δ*::imm434/ura3*Δ*::imm434*	(52)
*C. albicans*	RML2a	*ecm33∆*	*ecm33*Δ*::hisG/ecm33*Δ*::hisG ura3* Δ*::imm434/ura3*Δ*::imm434::URA3*	(53)
*C. albicans*	CM-1613C	*mkc1∆*	*mkc1*Δ *::hisG/mkc1Δ::hisG ura3Δ::imm434/ura3Δ::imm434*	(24)
*C. albicans*	*als4Δ*	*als4Δ*	*als4Δ::CaCas9 ura3Δ/ ura3Δ*	Malavia & Wilson, unpublished (D. Malavia Phd Thesis, University of Aberdeen)
*C. albicans*	CLF41	Clinical isolate	*GSC1 (F641S)/ GSC1 (F641S)*	Lass-Floerl, unpublished
*C. albicans*	CLF91	Clinical isolate	*GSC1 (S645F)/ GSC1 (S645F) GSC1wt/ GSC1 (R1361H)*	Lass-Floerl, unpublished
*C. albicans*	CLF32	Clinical isolate	*GSC1 (F641S)/ GSC1 (F641S)*	Lass-Floerl, unpublished
*C. albicans*	CLF11	Clinical isolate	*GSC1 (S645P)/ GSC1 (S645P)*	Lass-Floerl, unpublished
*C. albicans*	CLF52	Clinical isolate	*GSC1 (S645Y)/ GSC1 (S645Y)*	Lass-Floerl, unpublished
*C. albicans*	CLF82	Clinical isolate	*GSC1 (P649H)/ GSC1 (P649H)*	Lass-Floerl, unpublished
*C. albicans*	CLF403	Clinical isolate	*GSC1 (R1361S)/ GSC1 (R1361S)*	Lass-Floerl, unpublished
*C. albicans*	CLF71	Clinical isolate	*GSC1 (D648Y)/ GSC1 (D648Y)*	Lass-Floerl, unpublished
*C. albicans*	CLF61	Clinical isolate	*GSC1 (S645F)/ GSC1 (S645F)*	Lass-Floerl, unpublished
*C. albicans*	CLF435	Clinical isolate	*GSC1 (S645P)/ GSC1 (S645P)*	Lass-Floerl, unpublished
*C. albicans*	CLF539	Clinical isolate	*GSC1 (S645P)/ GSC1 (S645P)*	Lass-Floerl, unpublished
*C. albicans*	CLF2	Clinical isolate	*GSC1 (S645P)/ GSC1 (S645P)*	Lass-Floerl, unpublished
*C. dubliniensis*	CLF10	Clinical isolate	*GSC1 (S645P)/ GSC1 (S645P)*	Lass-Floerl, unpublished
*C. dubliniensis*	CLF49	Clinical isolate	*wild type*	Lass-Floerl, unpublished
*C. glabrata*	CLF13	Clinical isolate	*GSC1 (F625S)/ GSC1 (F625S)*	Lass-Floerl, unpublished
*C. glabrata*	CLF4	Clinical isolate	*FKS2 (F659S)/ FKS2 (F659S)*	Lass-Floerl, unpublished
*C. glabrata*	CLF34	Clinical isolate	*GSC1 (D632G)/ GSC1 (D632G)*	Lass-Floerl, unpublished
*C. glabrata*	CLF73	Clinical isolate	*GSC1 (D666G)/ GSC1 (D666G)*	Lass-Floerl, unpublished
*C. glabrata*	CLF24	Clinical isolate	*GSC1 (S629P)/ GSC1 (S629P)*	Lass-Floerl, unpublished
*C. glabrata*	CLF43	Clinical isolate	*GSC1 (F659V)/ GSC1 (F659V)*	Lass-Floerl, unpublished
*C. glabrata*	CLF84	Clinical isolate	*GSC1 (D666E)/ GSC1 (D666E)*	Lass-Floerl, unpublished
*C. glabrata*	CLF93	Clinical isolate	*GSC1 (P667T)/ GSC1 (P667T)*	Lass-Floerl, unpublished
*C. glabrata*	CLF83	Clinical isolate	*FKS2 (S663F)/ FKS2 (S663F)*	Lass-Floerl, unpublished
*C. glabrata*	CLF63	Clinical isolate	*GSC1 (S663P)/ GSC1 (S663P)*	Lass-Floerl, unpublished
*C. tropicalis*	CLF36	Clinical isolate	*GSC1 (F641S)/ GSC1 (F641S)*	Lass-Floerl, unpublished
*C. tropicalis*	CLF45	Clinical isolate	*wild type*	Lass-Floerl, unpublished
*C. tropicalis*	CLF75	Clinical isolate	*wild type*	Lass-Floerl, unpublished
*C. tropicalis*	CLF6	Clinical isolate	*GSC1 (S80P)/ GSC1 (S80P)*	Lass-Floerl, unpublished
*C. tropicalis*	CLF56	Clinical isolate	*wild type*	Lass-Floerl, unpublished
*C. tropicalis*	CLF86	Clinical isolate	*wild type*	Lass-Floerl, unpublished
*C. tropicalis*	CLF65	Clinical isolate	*GSC1 (V778I)/ GSC1 (V778I) GSC1 (V830I)/ GSC1 (V830I)*	Lass-Floerl, unpublished
*C. tropicalis*	CLF26	Clinical isolate	*GSC1wt/ GSC1 (S645P)*	Lass-Floerl, unpublished
*C. tropicalis*	CLF15	Clinical isolate	*GSC1wt/ GSC1 (S645P)*	Lass-Floerl, unpublished
*C. krusei*	CLF60	Clinical isolate	*GSC1 (D700M)/ GSC1 (D700M)*	Lass-Floerl, unpublished
*C. krusei*	CLF40	Clinical isolate	*GSC1wt/ GSC1 (F655C)*	Lass-Floerl, unpublished
*C. krusei*	CLF30	Clinical isolate	*GSC1 (R1361G)/ GSC1 (R1361G)*	Lass-Floerl, unpublished
*C. krusei*	CLF69	Clinical isolate	*wild type*	Lass-Floerl, unpublished
*C. krusei*	CLF79	Clinical isolate	*GSC1 (L701M)/ GSC1 (L701M)*	Lass-Floerl, unpublished
*C. parapsilosis*	CLF77	Clinical isolate	*wild type*	Lass-Floerl, unpublished
*C. parapsilosis*	CLF8	Clinical isolate	*wild type*	Lass-Floerl, unpublished
*C. parapsilosis*	CLF97	Clinical isolate	*wild type*	Lass-Floerl, unpublished
*C. parapsilosis*	CLF88	Clinical isolate	*wild type*	Lass-Floerl, unpublished
*C. parapsilosis*	CLF58	Clinical isolate	*wild type*	Lass-Floerl, unpublished
*C. parapsilosis*	CLF17	Clinical isolate	*wild type*	Lass-Floerl, unpublished
*C. parapsilosis*	CLF67	Clinical isolate	*wild type*	Lass-Floerl, unpublished
*C. parapsilosis*	CLF47	Clinical isolate	*wild type*	Lass-Floerl, unpublished
*C. lusitaniae*	CLF19	Clinical isolate	*wild type*	Lass-Floerl, unpublished
*E. coli*	TG1		*{SupE thi-1Δ (lac-proAB)Δ(mcrB-hsdSM)5 (rk –mk-) [F’traD36 proAB lacq ZM15]}*	QvQ

### Formaldehyde inactivation of *Candida* cells

This procedure was followed for the *C. albicans* cells used in all the experiments performed with the VHH antibody domains, unless otherwise specified. Cells were washed three times in PBS and incubated for 1 h at RT with 4% formaldehyde solution in 0.1M PHEM buffer (120 mM PIPES, 50 mM HEPES, 4 mM MgCl2*6H2O, 20 mM EGTA, pH 6.9) and stored overnight at 4°C. For further analysis, cells were thoroughly washed three times prior to use with PBS.

### Selection and validation of VHH binding to *C. albicans* whole cells

VHHs were selected using phage-display technology (20) and with the same strategy used for the anti-candidalysin VHH (21). To obtain VHHs able to bind to *C. albicans* cells, two *Llama glama* were immunized with heat-killed and formaldehyde-inactivated *C. albicans* cells (SC5314 strain) in different morphological states (i.e., yeast, hyphae, and germ tubes). After five boosts with similar numbers of cells, blood samples were taken to verify the immune response to *C. albicans* by ELISA. Lymphocytes were subsequently extracted, total RNA was isolated and converted into cDNA, and the VHH genes were cloned into pMEK222 phagemid to obtain DNA libraries (derived from pUR8100 phagemid, Fig. S1). For phage amplification and production, fresh *E. coli* TG1 cultures were grown with the DNA libraries in a selective medium. The bacteria were then infected with helper phage VCSM13, and phages were produced at 37°C overnight, before collecting the supernatants. The phages were precipitated using a 20% PEG6000/2.5M NaCl solution for 30 min at 4°C. With the phage libraries, two successive rounds of selection by bio-panning were performed on formaldehyde-inactivated whole cells and cell wall extracts from *C. albicans*. The first round was carried out by selecting the phage against cells from a pool of three caspofungin-resistant isolates of *C. albicans* (K063-3, B15_004476, B12_007355_1) described in the work from Buda De Cesare et al, (22). In the first round, bio-panning was performed against the pooled cells, untreated or treated with caspofungin (Table 1, column 1). isolates. The output from the first round was used for the second round of selection, this time with cell wall fractions or with fixed cells, both from the pooled caspofungin-resistant isolates. Cell wall fractions and fixed cells were prepared from cultures with or without caspofungin treatment (Table 1, column 2). As the aim was to isolate VHHs against caspofungin-resistant isolates, the output libraries were then counter selected against whole cells from a pool of three caspofungin-susceptible isolates (SC5314, ATCC76615, and B17_008835) described in the work from Buda De Cesare et al. (22). The specificity and the quality of the binding of the eluted phages were then verified by ELISA on whole fixed cells from the same pools of *C. albicans* isolates used in the first round of selection in yeast and hyphae. The selection of the VHHs was based on the strength and the pattern of the binding observed in the ELISA. The selected VHHs were sequenced using generic M13 primers provided by the sequencing service. VHHs with interesting sequences were cloned into *E. coli* expression plasmid pMEK222 (Fig. S1).

### VHH expression and purification in *E. coli*

The selected VHHs were expressed in *E. coli* cells and purified from the periplasmic fractions with IMAC according to Van Lith et al. (54). Briefly, exponential phase *E. coli* TG1 cells were grown in selective medium at 37°C, and expression was induced for 4 h with IPTG. The periplasmic protein fraction was isolated and then incubated with Talon beads (GE Healthcare) and eluted with imidazole. The collected fractions were then dialysed in 1X PBS for two rounds of 1 h at RT and one overnight at 4°C. The final concentration of the protein was measured at OD280 in a spectrophotometer, and the was quality verified by Sodium Dodecyl Sulphate-Polyacrylamide Gel Electrophoresis (SDS-PAGE).

### SDS-PAGE

VHH integrity and quality were verified by SDS-PAGE. A master mix composed of 100 mM dithiothreitol (DTT) reducing agent and NuPAGE LDS Sample Buffer (Invitrogen) was prepared. One microgram of the isolated VHH (based on the OD280 reading) was combined with the master mix and heated at 100°C for 10 min in a heating block. Samples were then loaded onto NuPAGE 4%–12% Bis-Tris Gel (1.0 mm × 15 well, Invitrogen), and the samples and standards were electrophoresed alongside 10 µL of PageRuler Prestained Protein Ladder (Thermo Fisher Scientific) and 1 µg of the reference VHH. The SDS–PAGE gel was run according to the manufacturer’s recommendations using 1× NuPAGE 3-(N-morpholino) propanesulfonic acid (MOPS) buffer. The gel was electrophoresed for 2 h at 40 mA and then stained for 1 h with PageBlue Protein Staining Solution (Thermo Fisher Scientific).

### Whole-cell ELISA

For assessing the binding capacity of the VHHs to the whole cells of *C. albicans*, formaldehyde-inactivated cells were used as described previously. Approximately 10^6^ cells/mL were used to coat the wells of a 96-well plate at RT for 2 h or at 4°C overnight. A 4% dried skimmed milk powder (Marvel) solution in PBS (MPBS) was used for blocking, and serial dilutions of the VHHs were added in the next step. Rabbit anti-VHH polyclonal antibody (QVQ) was used as secondary antibody and a donkey anti-rabbit IgG (H + L)–horseradish peroxidase–conjugate antibody (Jackson Immunoresearch) was added for detection with 3,3',5,5′-Tetramethylbenzidine (TMB, Thermo Fisher Scientific) substrate. The absorbance was measured at 655 nm in a VersaMax microplate reader (Molecular Devices, USA).

### Immunolabeling

In order to investigate the binding patterns of the VHHs to different cell morphologies and cell wall mutants, formaldehyde-fixed cells of *C. albicans* were blocked using a 0.5% solution of Bovine Serum Albumin fraction V (BSA, Roche Diagnostics) in PBS for 1 h at RT. After blocking, the cells were incubated with the different VHHs (1 µM dilutions) for 1 h at RT. The cells were then washed three times with PBS and incubated with a solution of rabbit anti-VHH polyclonal antibody (QVQ) in PBS with 0.5% BSA for 1 h at RT. The cells were washed three times in PBS and incubated with a Goat anti-Rabbit IgG (H + L)–Alexa Fluor 647–conjugated antibody (Invitrogen) for 1 h at RT. The cells were then washed three times and stained with 50 µg/mL calcofluor white (Fluorescent Brightener 28, Sigma–Aldrich Co.) in order to make the cell wall visible, and cells were imaged using a Zeiss Axio Imager M2 microscope equipped with a Zeiss 503 camera. For flow cytometry, the cells were prepared using the same protocol and analyzed with the Attune NxT Flow Cytometer (Invitrogen). For each sample, 10,000 events were collected, and the MFI was calculated using FlowJo software (BD Biosciences, USA).

## SUPPLEMENTAL MATERIAL

Fig. S1 spectrum.04269-23-s0001.tif Plasmid map.

Fig. S2 spectrum.04269-23-s0002.tif Master plate layout of ELISA with periplasmic extracts containing VHHs.

Fig. S3 spectrum.04269-23-s0003.tif Negative controls for fluorescence microscopy experiments with VHHs.

Fig. S4 spectrum.04269-23-s0004.tif Immunolabeling of *C. albicans* hyphae with VHH9.

Fig. S5 spectrum.04269-23-s0005.tif Immunolabeling of *C. albicans* hyphae with VHH2

Fig. S6 spectrum.04269-23-s0006.tif Immunolabeling of *C. albicans* als3Δ + ALS3 reintegrant hyphae with VHH14.

Supplemental material spectrum.04269-23-s0007.docx Supplemental figure legends.

ASM does not own the copyrights to Supplemental Material that may be linked to, or accessed through, an article. The authors have granted ASM a non-exclusive, world-wide license to publish the Supplemental Material files. Please contact the corresponding author directly for reuse.
