# Supplementary figures and images for: The development of single-domain VHH nanobodies that target the Candida albicans cell surface
Source: Microbiol Spectr. 2024 Oct 7;12(11):e04269-23. doi: 10.1128/spectrum.04269-23 (PMC11572700; doi:10.1128/spectrum.04269-23)

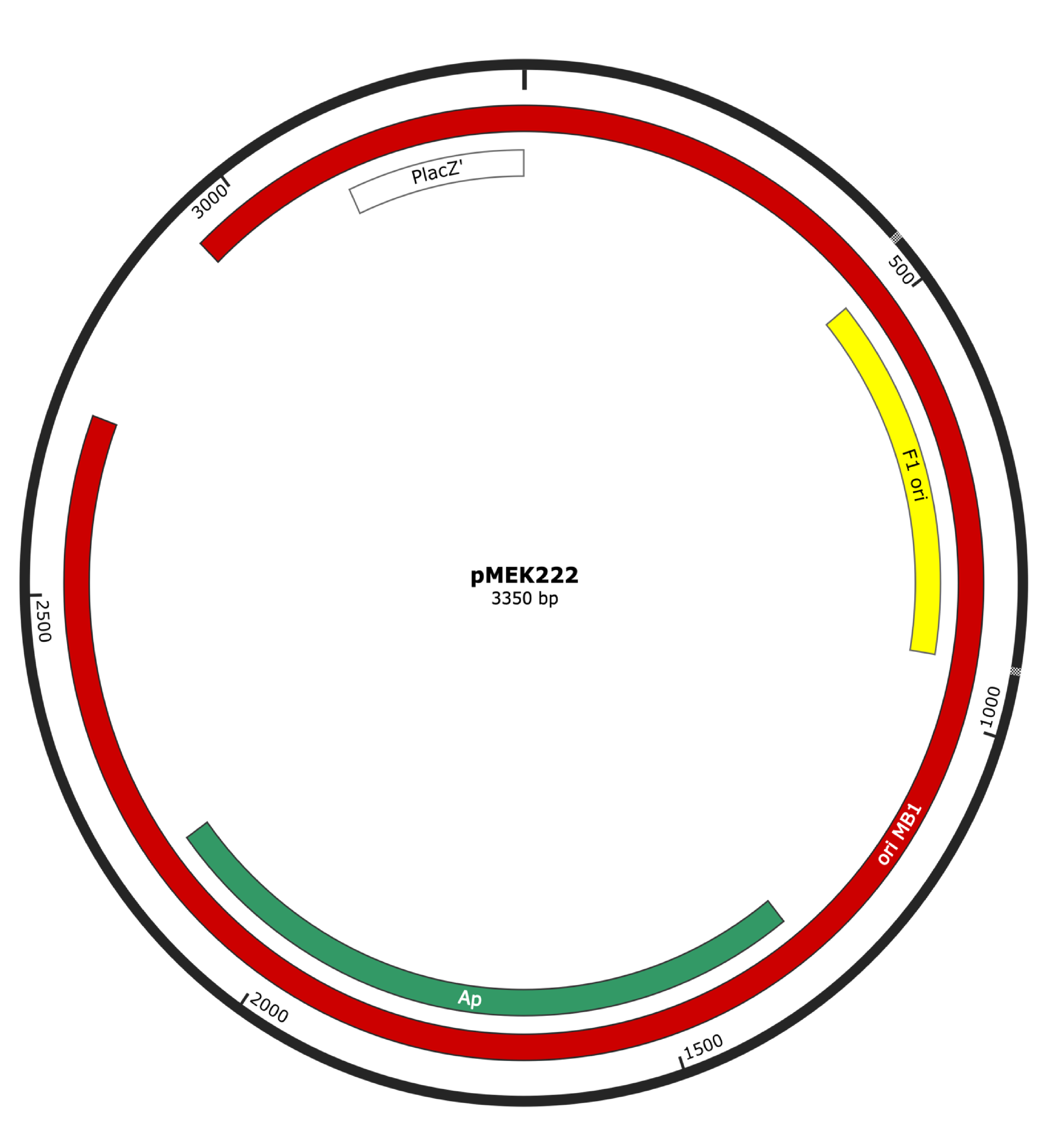

Supplement: Fig. S1 — Plasmid map. [file spectrum.04269-23-s0001.tif]

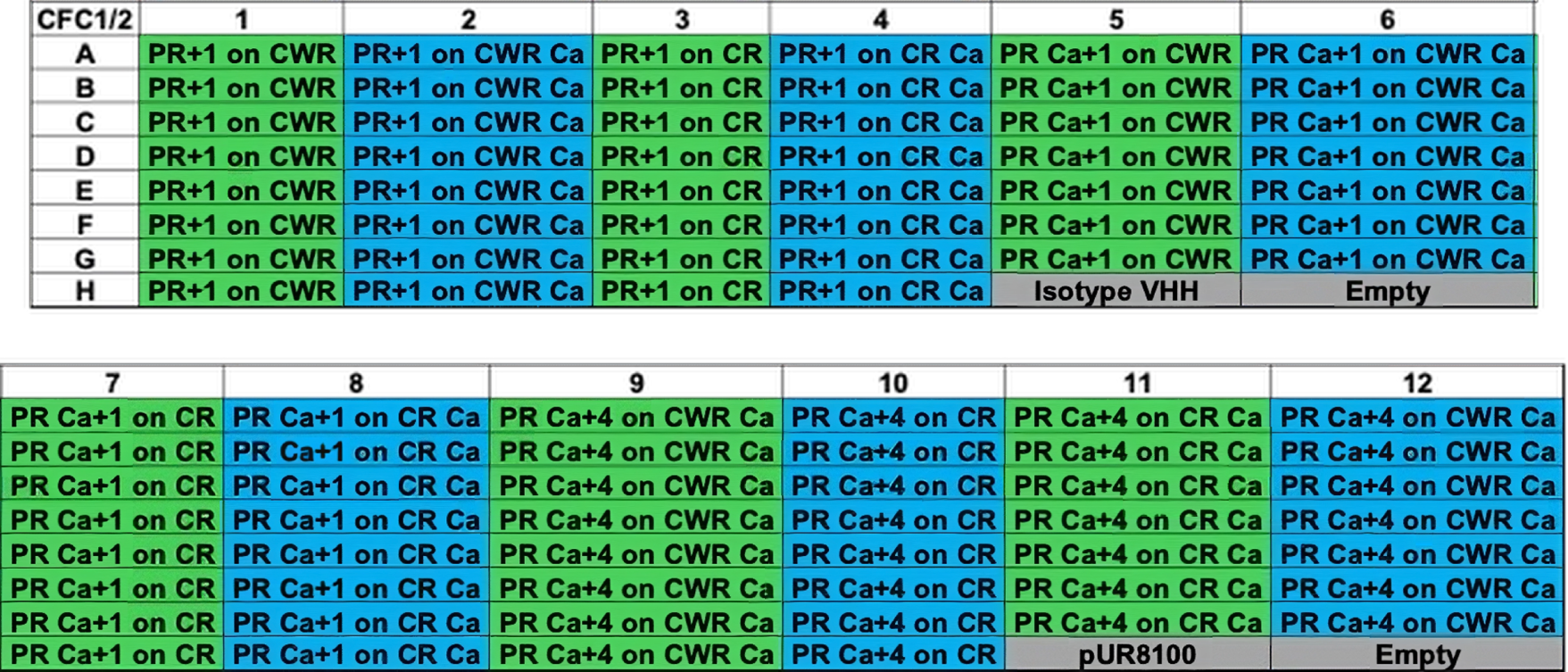

Supplement: Fig. S2 — Master plate layout of ELISA with periplasmic extracts containing VHHs. [file spectrum.04269-23-s0002.tif]

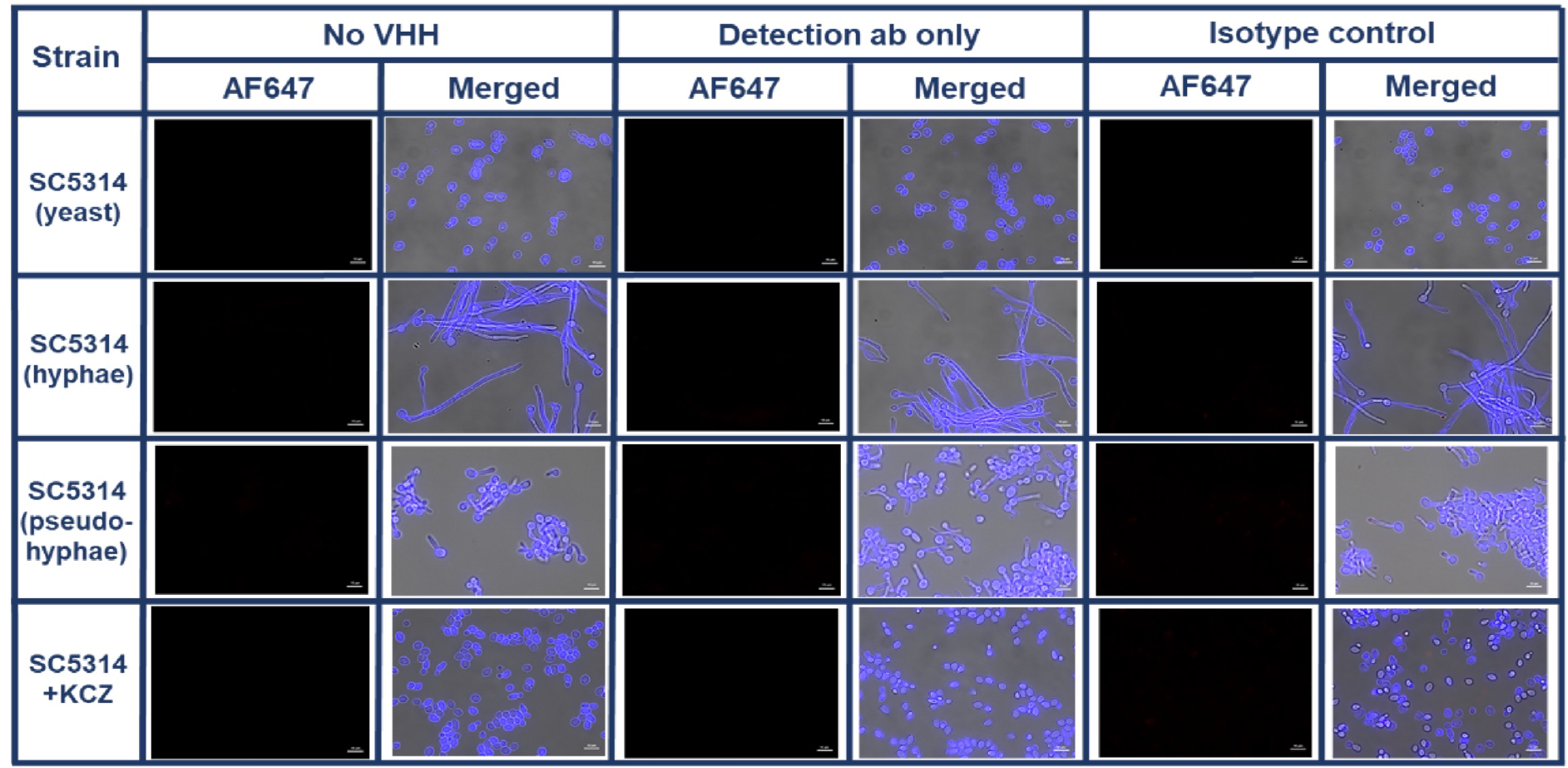

Supplement: Fig. S3 — Negative controls for fluorescence microscopy experiments with VHHs. [file spectrum.04269-23-s0003.tif]

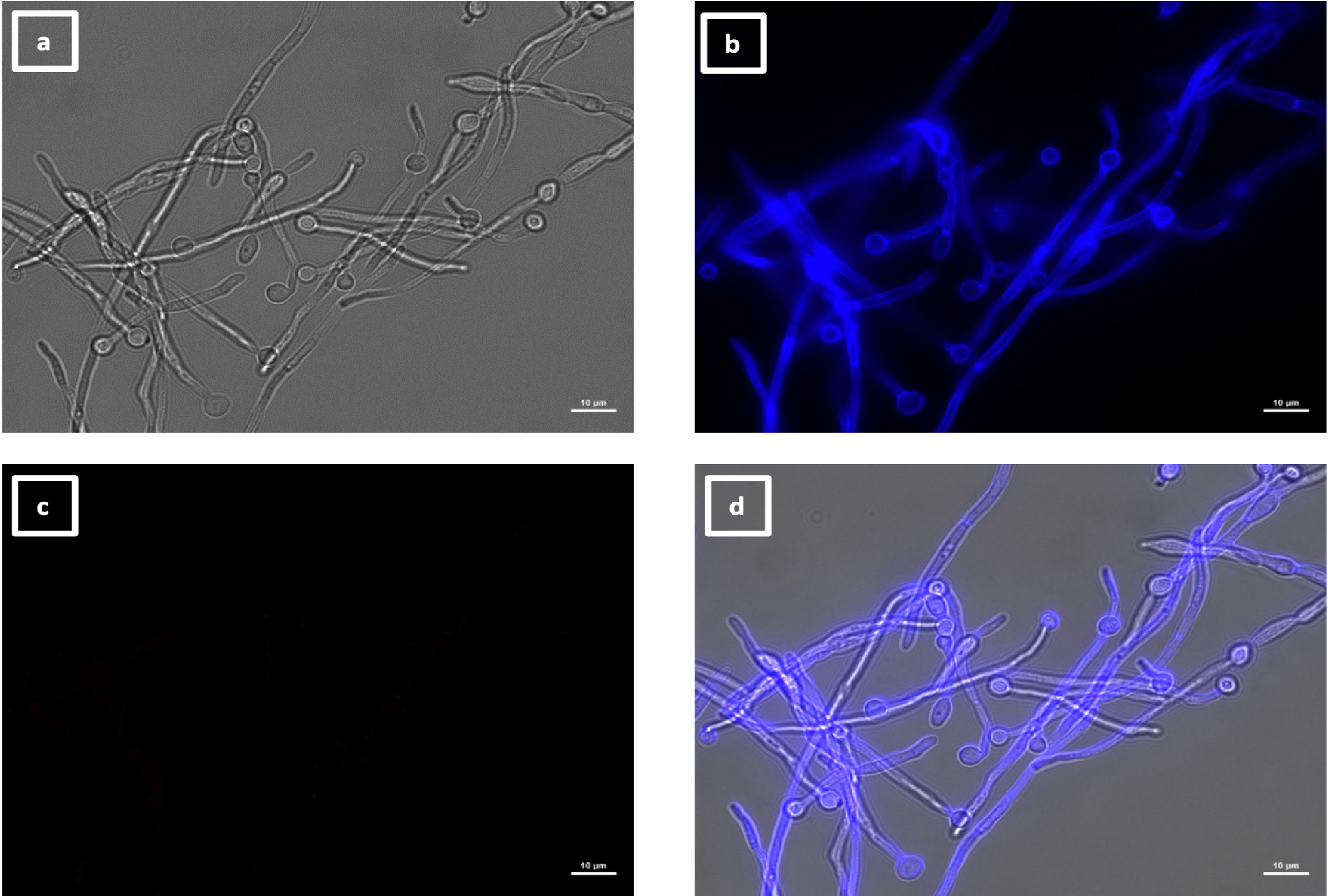

Supplement: Fig. S4 — Immunolabeling of C. albicans hyphae with VHH9. [file spectrum.04269-23-s0004.tif]

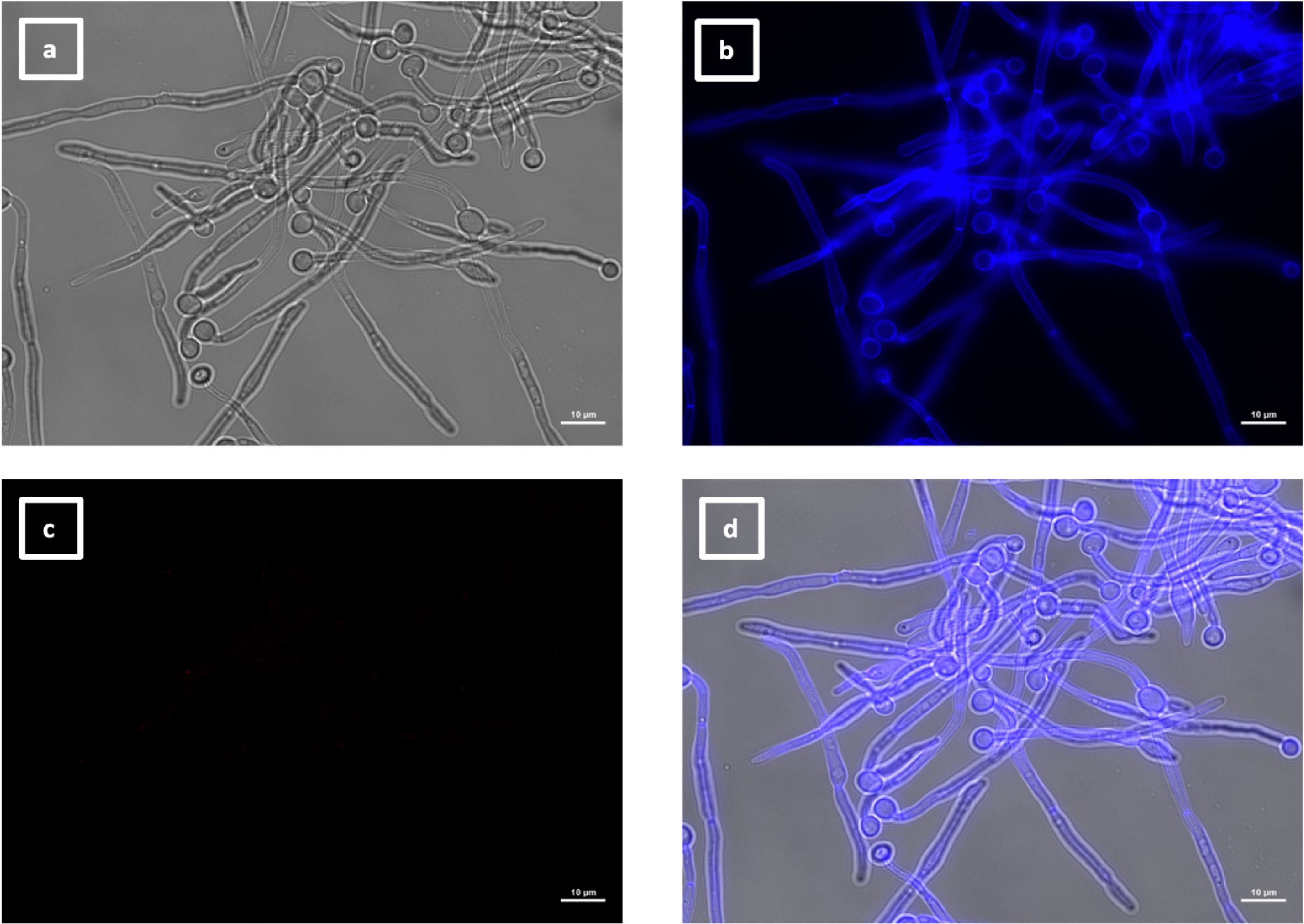

Supplement: Fig. S5 — Immunolabeling of C. albicans hyphae with VHH2 [file spectrum.04269-23-s0005.tif]

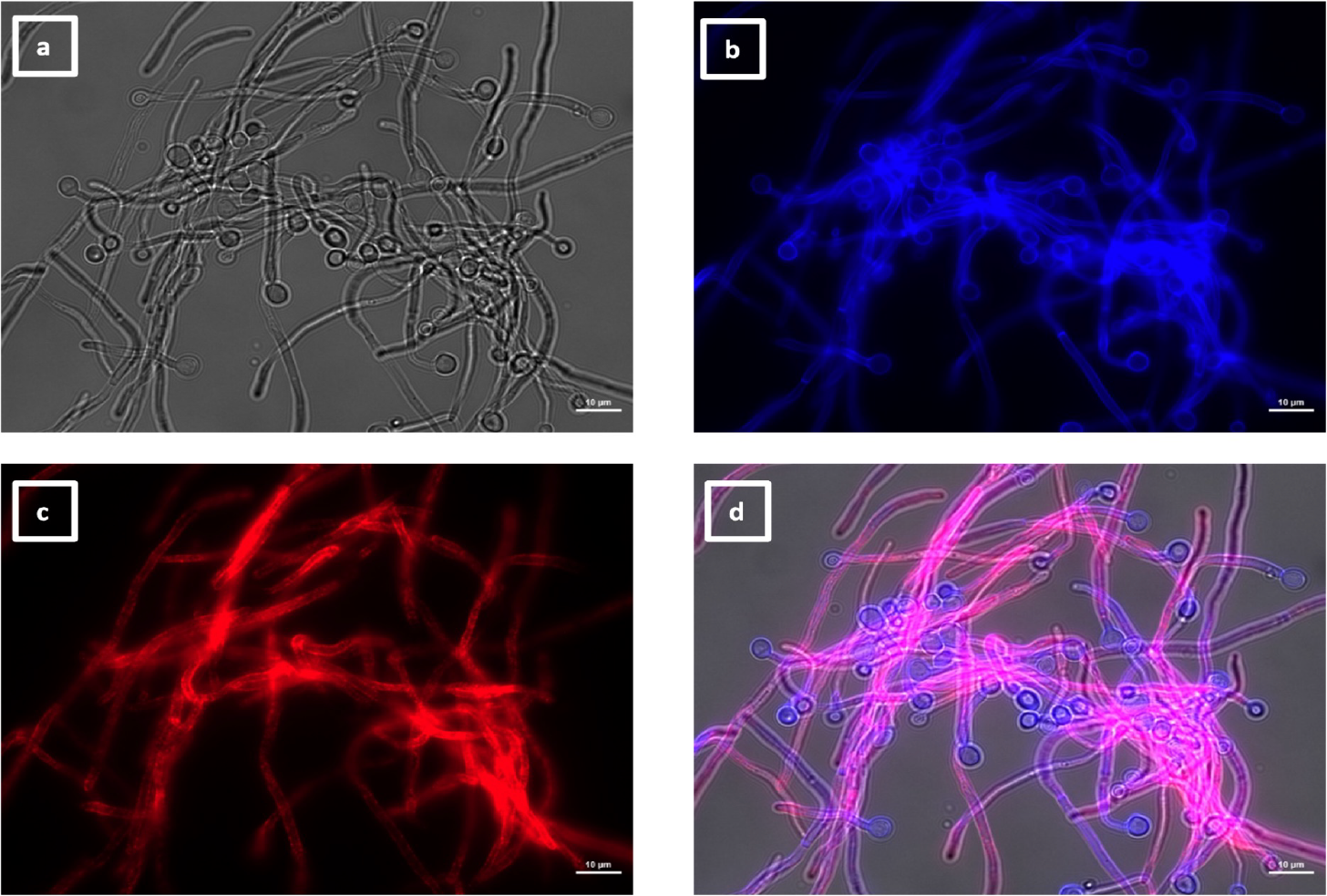

Supplement: Fig. S6 — Immunolabeling of C. albicans als3Δ + ALS3 reintegrant hyphae with VHH14. [file spectrum.04269-23-s0006.tif]
