# Supplementary material for: The development of single-domain VHH nanobodies that target the Candida albicans cell surface
Source: Microbiol Spectr. 2024 Oct 7;12(11):e04269-23. doi: 10.1128/spectrum.04269-23 (PMC11572700; doi:10.1128/spectrum.04269-23)
Supplement: Supplemental material — Supplemental figure legends. [file spectrum.04269-23-s0007.docx]

**Supplementary material**

**Figure S1. Plasmid used in this study for phage display and VHH purification.** pMEK222 used for VHH expression and purification.

**Figure S2. Master plate layout of ELISA with periplasmic extracts containing VHHs.** Each well contains a different periplasmic fraction derived from the phage libraries. The periplasmic extracts were assessed for binding to a pool of caspofungin-resistant isolates of *C. albicans* (4#). The clones were previously counter-selected against a pool of caspofungin-susceptible isolates of *C. albicans* (1). Whole cells (cells R) or cell wall extracts (CW R) were used for the experiment, of cells either treated with (Ca) or without caspofungin. The “+” symbol indicates the counterselection. The pool of caspofungin-resistant isolates includes: K063-3, B15_004476, B12_0073551, PEG10_91 and B15_012740. The pool of caspofungin-susceptible isolates includes: B17_008835, SC5314 and ATCC76615. This layout was used for both CFC-1 and CFC-2 plates displayed in Figure 1.

**Figure S3. Negative controls for fluorescence microscopy experiments with VHHs.** The samples were treated either without VHH (no VHH), without the secondary antibody (detection ab only) or with an VHH specific for a HIV protein (isotype control). The panel Alexa Fluor 647 (AF647) shows the signal derived from binding of the antibodies. The merged panel shows the merged view of all the channels used: DIC, calcofluor white (blue) and AF647.

**Figure S4. Immunolabelling of *C. albicans* hyphae with VHH9.** Hyphae cells of *C. albicans* strain SC5314 were grown in RPMI-1640 medium at 37ºC for 6 h and fixed with formaldehyde. The samples were stained with calcofluor white and labelled with VHH9, and a rabbit anti-VHH polyclonal antibody and a Goat anti-Rabbit IgG – Alexa Fluor 647 – conjugated antibody for detection. Panels indicate: (a) DIC; (b) calcofluor white; (c) Alexa Fluor 647; (d) merged view. Cells were imaged using a Zeiss Axio Imager M2 microscope.

**Figure S5. Immunolabelling of *C. albicans* hyphae with VHH2.** Hyphae of *C. albicans* strain SC5314 were grown in RPMI-1640 medium at 37ºC for 6 h and fixed with formaldehyde. The samples were stained with calcofluor white and labelled with VHH2, and a rabbit anti-VHH polyclonal antibody and a Goat anti-Rabbit IgG – Alexa Fluor 647 – conjugated antibody for detection. Panels indicate: (a) DIC; (b) calcofluor white; (c) Alexa Fluor 647; (d) merged view. Cells were imaged using a Zeiss Axio Imager M2 microscope.

**Figure S6. Immunolabelling of *C. albicans* *als3*Δ *+ ALS3* reintegrant hyphae with VHH14.** Hyphae of *C. albicans* *als3*Δ *+ ALS3* reintegrant strain were grown in RPMI-1640 medium at 37ºC for 6 h and fixed with formaldehyde. The samples were stained with calcofluor white and labelled with VHH14, and a rabbit anti-VHH polyclonal antibody and a Goat anti-Rabbit IgG – Alexa Fluor 647 – conjugated antibody for detection. Panels indicate: (a) DIC; (b) calcofluor white; (c) Alexa Fluor 647; (d) merged view. Cells were imaged using a Zeiss Axio Imager M2 microscope.
